# Methaneseleninic acid, a circadian-modulating agent, reactivates latent HIV-1 infection without cellular activation or proliferation

**DOI:** 10.1128/jvi.01983-25

**Published:** 2026-02-25

**Authors:** Jared Stern, Rory A. Shepherd, Youry Kim, Carolin Tumpach, Kirsten E. Amos, Oscar H. Lloyd Williams, Swati Varshney, Hannah A. D. King, Ajantha Rhodes, Sharon R. Lewin, Michael Roche

**Affiliations:** 1 Department of Infectious Diseases, The University of Melbourne at the Peter Doherty Institute for Infection and Immunity 534133 , Melbourne, Australia; 2 Melbourne Mass Spectrometry and Proteomics Facility, Bio21 Molecular Science and Biotechnology Institute, The University of Melbourne 195117, Melbourne, Australia; 3 Department of Infectious Diseases, Monash University and Alfred Hospital 589641, Melbourne, Victoria, Australia; 4 Victorian Infectious Diseases Service, Royal Melbourne Hospital at the Peter Doherty Institute for Infection and Immunity 534133 , Melbourne, Australia; 5 School of Health and Biomedical Sciences, RMIT University 213179, Melbourne, Australia; University Hospital Tübingen, Tübingen, Germany

**Keywords:** selenium, latency reversal, HIV, circadian, T-cell proliferation

## Abstract

Human immunodeficiency virus (HIV)-1 transcriptional activity is under circadian control in virally suppressed people with HIV (PWH), and circadian transcription factors can positively affect HIV transcription *in vitro*. We aimed to determine whether pharmacologic modulation of cell-autonomous circadian cycles can reverse HIV latency. We examined the effects of a known circadian modulator, the organic selenium compound, methaneseleninic acid (MSA), on viral reactivation, and cellular activation and proliferation in near-full-length latently infected cell lines and primary peripheral blood CD4+ T cells from virally suppressed males with HIV. MSA robustly reactivated HIV in latently infected cell lines and induced an increase in cell-associated unspliced but not multiply spliced HIV RNA in primary CD4+ T cells without cellular activation, proliferation, or changes in HIV DNA. Bulk RNA sequencing of primary CD4+ T cells from people with HIV indicated MSA alters gene expression favoring apoptosis. This work promisingly demonstrates a novel target to reactivate latent HIV.

**IMPORTANCE:**

This study explores a novel and promising approach to tackle HIV latency, a major barrier to curing HIV infection. Despite effective antiretroviral therapy, HIV can persist in a dormant state within certain cells, forming a reservoir that is challenging to eliminate. This research investigates the potential of modulating the body’s internal circadian rhythms, which regulate many biological processes, as a means to reactivate and purge this latent HIV reservoir. Specifically, it was found that a circadian-modulating compound called methaneseleninic acid could robustly reactivate latent HIV in infected cell lines and primary cells from people with HIV, without causing broader cellular activation or proliferation. This targeted reactivation of latent HIV by manipulating circadian cycles represents a unique and intriguing strategy that could potentially contribute to future HIV cure efforts.

## INTRODUCTION

Antiretroviral therapy (ART) has significantly advanced treatment for people with HIV-1 (PWH) to reduce morbidity, mortality, and onward transmission. However, ART cannot cure HIV, and treatment needs to be taken lifelong (1). The major barrier to a cure for HIV-1 is the persistence of a viral reservoir comprised of long-lived and replicating infected cells that harbor an integrated, intact, replication-competent HIV-1 provirus capable of viral rebound upon ART cessation (2, 3). One strategy being examined to clear the latent, replication-competent reservoir is to induce viral transcription, antigen expression, and drive apoptosis sufficient for the clearance of infected cells (4–7).

The majority of infected cells on ART are latent, with proviruses that are entirely quiescent or express minimal levels of cell-associated HIV RNA without enough surface antigen expression for adequate immune recognition and clearance (8–11). Moreover, latently infected cells are recalcitrant to apoptosis, and the reservoir is maintained in a quasi-equilibrium due to ongoing cellular proliferation in response to antigen and circulating cytokines (12–17). The state of HIV latency is dynamic; transcriptional and reactivation potential are dependent on the site and orientation of proviral integration and its subsequent epigenetic remodeling (18–21); viral activation can be decoupled from cellular activation (8, 22, 23); and reactivation from latency occurs as stochastic bursts which can lead to either productive infection or a reversion to latency (24–26). Furthermore, we recently demonstrated that HIV RNA transcription in PWH on ART varies with a circadian rhythm (27). We therefore asked whether modulation of proteins that regulate circadian rhythms could be exploited to reverse HIV latency.

The two major cellular transcription factors in the cell-autonomous circadian cycle, Circadian Locomotor Output Cycles Kaput (CLOCK) and Brain and Muscle ARNT-Like 1 (BMAL1), have been shown to positively regulate HIV transcription *in vitro* (28, 29). CLOCK and BMAL1 can heterodimerize and bind to short palindromic DNA motifs called “E-boxes” within gene promoters to initiate transcription of clock-controlled genes (30), of which the HIV-1 5′ long terminal repeat (LTR) contains four putative E-boxes (31). We and others have demonstrated that the second E-box motif, upstream of the NF-κB-binding sites, is crucially and solely required for CLOCK:BMAL1’s ability to induce HIV transcription *in vitro* (28, 29).

Two gene families under CLOCK:BMAL1 control are *Period* (*PER1, 2,* and *3*) and *Cryptochrome* (*CRY1* and *2*), which make up the negative feedback arm of the circadian cycle by directly inhibiting CLOCK and/or BMAL1 function. PER and CRY, in inhibiting their own production to eventually release CLOCK and BMAL1 from their repression, create the cyclical nature of the circadian feedback loop. Further fine-tuning of this cycle occurs through additional transcription factors such as REV-ERBα/β and RORα/β, which regulate *BMAL1* transcription by binding to retinoic acid-related orphan receptor response elements (ROREs) found within the promoter of *BMAL1 (32*). The HIV-1 5′ LTR also contains ROREs, and ROR inhibition precludes viral transcription, further implicating a circadian influence on HIV-1 activity (33).

We hypothesized that inhibition or activation of proteins that regulate the circadian rhythm could potentially be exploited as latency-reversing agents. Here, we demonstrate that pharmacologic activation of *BMAL1* transcription with an organic selenium compound, methaneseleninic acid (MSA), led to latency reversal in latently infected cell lines and initiation of HIV transcription in primary CD4+ T cells from virally suppressed cisgender males with HIV-1. Our work highlights a druggable cell-intrinsic pathway to target for HIV-1 latency reversal with a promising safety profile that could potentially be tested in clinical trials.

## RESULTS

### MSA induces both HIV expression and *BMAL1* transcription in latently infected T-cell lines

**Fig 1:**
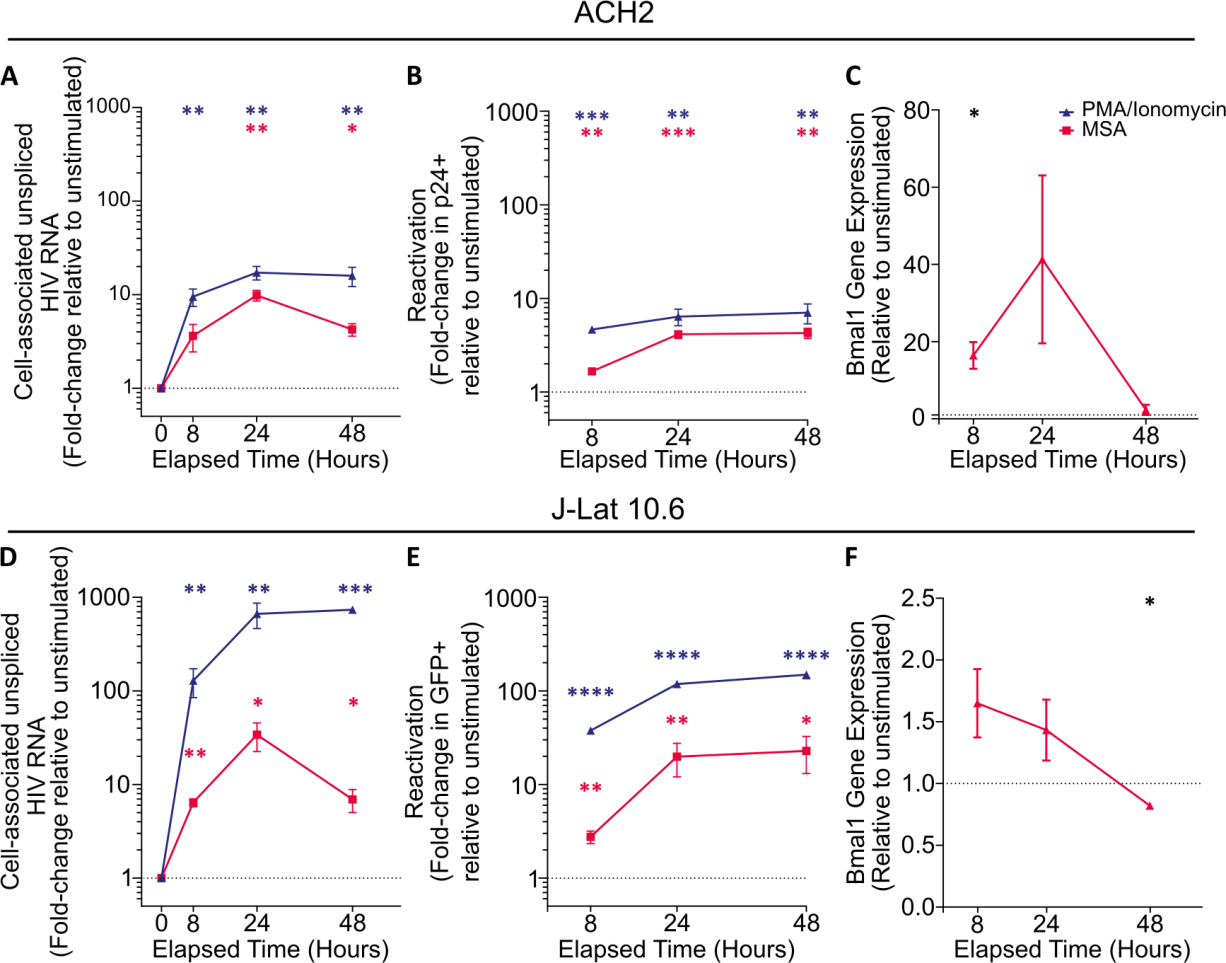
MSA reactivates HIV-1 and induces BMAL1 transcription in latently infected T-cell lines. ACH2 and J-Lat 10.6 cells were stimulated with 10 μM MSA (red) for 18 h, at which point the drug was washed off and cells were cultured for a further 30 h. Cells left unstimulated (black) or stimulated with 16 nM phorbol 12-myristate 13-acetate (PMA) and 500 μM ionomycin (blue) provided negative and positive controls for viral reactivation, respectively. Cell-associated unspliced HIV-1 RNA (**A, D**) was measured by RT-qPCR. Viral reactivation—as measured by GFP or p24 expression (**B, E**)—was assessed by flow cytometry. BMAL1 gene expression was measured by RT-qPCR and fold-changes calculated compared to baseline using the ΔΔCt relative quantification method (**B, F**). Fold-changes were calculated relative to unstimulated cells at 0 h (**A, D**) or 8 h (**B, C, E, F**). *n* = 3 (**A, D**) or 4 (**B, E**), datapoints represent the mean ± SEM. Statistical significance was calculated by paired t-tests; *P* < 0.05, *; *P* < 0.01, **; *P* < 0.001, ***; *P* < 0.0001, ****.

Given our observations of circadian rhythmicity in HIV transcription *in vivo* (27) and CLOCK:BMAL1’s initiation of viral transcription *in vitro* (28), we examined whether pharmacologic modulation of cell-autonomous circadian cycles could act as latency reversal agents (LRAs). Resveratrol, nobiletin, ivermectin, MSA, and methylselenocysteine (MSC) were identified in the literature to modulate circadian machinery (summarized in Table S1) and were titrated on the T-cell line, J-Lat Tat-IRES-GFP clone A2 containing a minimal HIV reporter integrant to assess viral reactivation. The organic selenium compounds, MSC and MSA, which increase *BMAL1* transcription *in vitro* (34), demonstrated the greatest reactivation out of the compounds tested, and MSA had the most favorable therapeutic window (Table S1; Fig. S1). We next proceeded to test MSA’s LRA properties in the more relevant T-cell lines, J-Lat 10.6, and ACH2, which harbor integrated near full-length and full-length intact HIV-1 proviruses, respectively, but with varied integration sites and parental cell origin to provide some understanding of reactivation breadth. Following MSA treatment, cell-associated unspliced (CA-US) HIV RNA—a full-length transcript indicative of transcriptional initiation—in J-Lat 10.6 and ACH2 cells increased early within 8 h (6.44 ± 0.66 fold [*P* = 0.0031] and 3.63 ± 1.18 fold [*P* = 0.058], respectively) before peaking by 24 h (33.97 ± 11.5 fold [*P* = 0.019] and 9.82 ± 1.28 fold [*P* = 0.0031], respectively) (Fig. 1A and D; Fig. S3B and I). CA-US HIV RNA decreased from their peak but remained elevated for 48 h in both J-Lat 10.6 and ACH2 cells (6.92 ± 1.91 fold [*P* = 0.035] and 4.24 ± 0.64 fold [*P* = 0.011], respectively), despite MSA being removed from culture 18 h post-stimulation.

Intracellular HIV protein expression (as represented by GFP in J-Lat 10.6 and HIV capsid, p24, in ACH2 cells) followed similar dynamics to that of CA-US HIV RNA, with protein expression increasing as early as 8 h after MSA stimulation before reaching sustained peak expression later (22.84 ± 9.77 fold [*P* = 0.016] and 4.14 ± 0.41 fold [*P* = 0.00084], respectively) (Fig. 1B and E; Fig. S3C and J). The lower magnitude increases seen in ACH2 cells compared to J-Lat 10.6 cells reflect these cells’ higher HIV expression at baseline—indeed, a higher frequency of ACH2 cells was p24+ with MSA stimulation compared to J-Lat 10.6 cells expressing GFP (Fig. S3C and J).

In addition to upregulating HIV expression in these latently infected cell lines, MSA quickly and potently increased *BMAL1* levels. In ACH2 cells, *BMAL1* mRNA increased 16.43 ± 3.47 fold (*P* = 0.021) at 8 h, peaked by 24 h (41.3 ± 21.75 fold, *P* = 0.160) and returned to baseline by 48 h (Fig. 1C). Though of lower magnitude induction, MSA trended to increase *BMAL1* mRNA in J-Lat 10.6 cells, where a peak was observed as early as 8 h (1.65 ± 0.28 fold, *P* > 0.1) before beginning to decrease by 48 h (Fig. 1F). There was a non-significant increase in *PER* expression at later timepoints (Fig. S3). These data indicate that MSA quickly increases *BMAL1* mRNA as well as HIV transcription that results in robust HIV protein expression, even after MSA was removed from cultures.

### HIV RNA initiation and *BMAL1* are upregulated by MSA in primary CD4+ T cells from PWH

To confirm the effects of circadian modulation on HIV transcription by MSA, primary peripheral blood CD4+ T cells from eight virally suppressed white cisgender males with HIV were cultured with 10 μM MSA for 24 h prior to drug washout and continued culture up to 72 h post-stimulation. MSA stimulation had favorable toxicity in primary CD4+ T cells compared to the T-cell lines (median 77.2% viability at 72 h, compared to 85.1% for unstimulated *P* = 0.008) (Fig. S5A).

**Fig 2:**
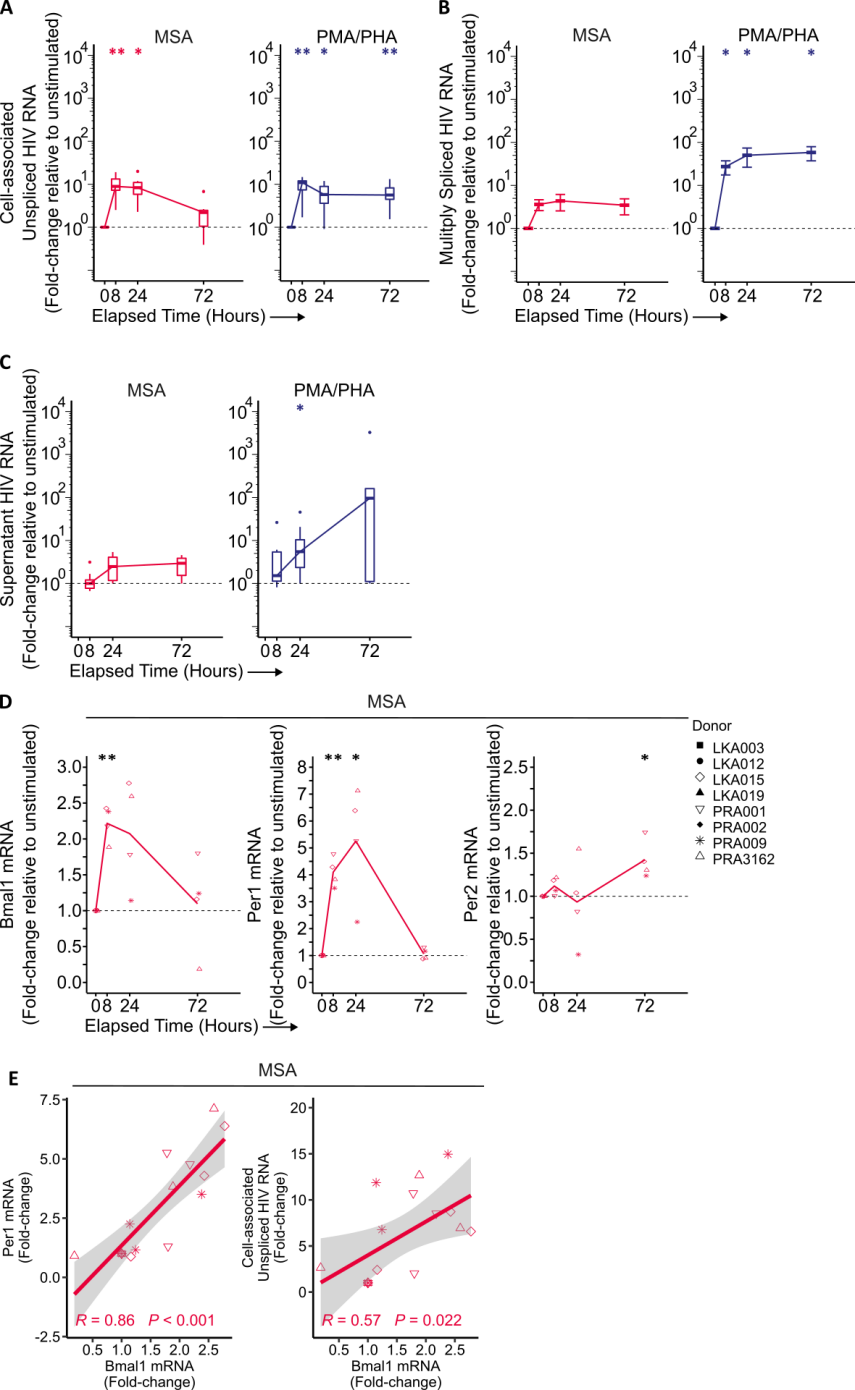
MSA potently induces the expression of cell-associated unspliced HIV-1 and BMAL1 RNA in cells from PWH *ex vivo*. CD4+ T cells were isolated from PBMCs from PWH and cultured in the presence of 10 μM MSA (red) for 24 h before drug wash off, or continuous culture with 10 nM PMA and 10 μg/mL phytohemagglutinin (PHA) (blue). Cell-associated unspliced (**A**) and multiply spliced (**B**) HIV-1 RNA was quantified from bulk RNA, and the supernatant HIV-1 RNA (**C**) was quantified by RT-qPCR. Fold-changes are relative to baseline expression prior to stimulation (0 h). Circadian gene expression (**D**) was quantified by RT-qPCR, relative to the reference gene B2M. Fold-changes were calculated relative to baseline expression at 0 h. (**A, C**) *n* = 8 independent donors, boxplots represent the median and interquartile range, whiskers represent the minima and maxima, and datapoints represent outliers. Statistical comparison to unstimulated cells was determined using a Wilcoxon matched-pairs signed rank test; *P* < 0.05, *; *P* < 0.01, **. (**B, D**) *n* = 4–6 independent donors, lines represent the mean, and datapoints represent each participant. Statistical significance was determined using paired t-tests compared to baseline (onefold); *P* < 0.05, *; *P* < 0.01, **. (**E**) Correlation plots of *Bmal1* mRNA with *Per1* (left) or CA-US HIV (right) RNA levels using a linear model; R, Pearson correlation; *P*, *P*-value.

Similar to the cell lines, CA-US HIV RNA was quickly upregulated in primary CD4+ T cells, reaching a peak 8.9-fold [4.7- to 14.3-fold] increase by 8 h from baseline (*P* = 0.008) and remained elevated out to 24 h before beginning to return to baseline by 72 h (Fig. 2A; Fig. S5B). The peak fold-increase in CA-US HIV RNA with MSA stimulation was comparable to the increases achieved with a maximal TCR-stimulus using PMA/PHA (*P* = 0.8 comparing PMA/PHA to MSA at 8 h). Multiply spliced (MS) HIV RNA, a key step in virus replication for expression of HIV envelope and accessory proteins (35), did not significantly increase with MSA stimulation (Fig. 2B; Fig. S5C), nor did supernatant HIV RNA—a measure of cell-free virion release (Fig. 2C; Fig. S5D). As expected, maximal TCR stimulation with PMA/PHA robustly induced MS HIV RNA transcripts, with a modest increase in virion release later at 72 h. CA-US transcripts were modestly positively correlated with multiple spliced transcripts after MSA and PMA/PHA stimulation (Fig. S5E), but neither transcript was correlated with supernatant HIV virion release (Fig. S5F and G).

*BMAL1* mRNA, like the early HIV RNA transcripts, was quickly elevated upon MSA stimulation, reaching a peak 2.22 ± 0.25 fold above baseline (*P* = 0.002) within 8 h (Fig. 2D, left). There was a non-significant 2.07 ± 0.76 fold (*P* = 0.066) increase in *BMAL1* at 24 h before returning to baseline by 72 h. The repressor gene, *PER1*, demonstrated similar kinetics with MSA stimulation; an early 4.1 ± 0.55 fold (*P* = 0.0015) increase at 8 h, peaking at 24 h (5.26 ± 2.15 fold) before returning to baseline by 72 h (Fig. 2D, middle). Perturbations to *PER2* expression were less pronounced, which remained near baseline expression in the first 24 h before being slightly increased 1.42 ± 0.23 fold (*P* = 0.033) by 72 h (Fig. 2D, right). Moreover, upon MSA stimulation, mRNA levels of *BMAL1* were strongly positively correlated with both *PER1* (R = 0.86, *P* < 0.001, Fig. 2E, left) and CA-US HIV RNA (R = 0.57, *P* = 0.022, Fig. 2E, right).

Together, these data confirm MSA’s ability to increase both *BMAL1* and early HIV RNA expression in CD4+ T cells from virally suppressed PWH *ex vivo*. In line with MSA’s mechanism of action on the HIV LTR, MSA did not induce spliced HIV RNA nor virion release.

### MSA reduces CD38 expression in primary CD4+ T cells from PWH

Although global T-cell activation can lead to pronounced latent HIV reactivation, it is preferable that latency reversal does not require cellular activation, to avoid broad, chronic T-cell activation and a potential systemic cytokine storm *in vivo* (36). Thus, we measured early (CD69) and late (CD38, HLA-DR) T-cell activation in response to MSA stimulation in primary CD4+ T cells from PWH.

**Fig 3:**
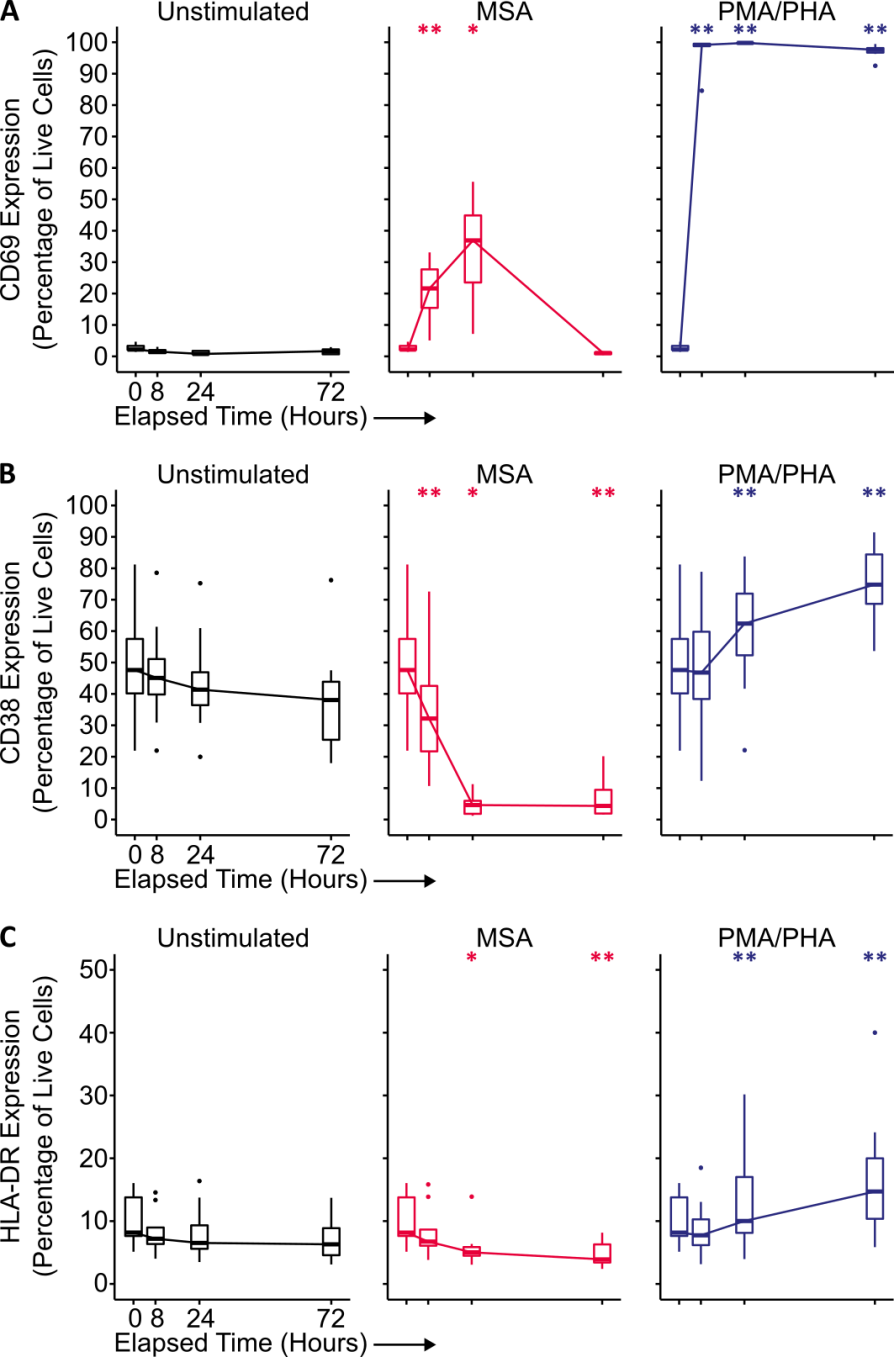
MSA induces transient CD69 activation while decreasing CD38 expression in CD4+ T cells from PWH. CD4+ T cells were isolated from PBMCs from PWH and cultured in the presence of 10 μM MSA (red) for 24 h before drug wash off, or continuous culture with 10 nM PMA and 10 μg/mL PHA (blue). Surface expression of early (CD69; **A**) and late activation markers (CD38, HLA-DR; **B and C**) was assessed by flow cytometry. *n* = 8 independent donors, boxplots represent the median, 25th and 75th percentiles, whiskers represent the minima and maxima, and datapoints represent outliers. Statistical significance was determined using the Wilcoxon matched-pairs signed rank test; *P* < 0.05, *; *P* < 0.01, **.

With similar kinetics to the induction of CA-US HIV RNA, CD69 expression was increased as early as 8 h, peaking at 24 h with 36.9% (7.2%–55.6%, *P* = 0.016 compared to unstimulated cells) CD69+ CD4+ T cells, before returning to baseline by 72 h (Fig. 3A, middle). Unexpectedly, MSA potently decreased the late activation marker, CD38, from a median of 47.6% (21.9%–81.2%) at baseline to 4.6% (1.2%–11.3 %) by 24 h (*P* = 0.016) (Fig. 3B, middle). Unlike CD69 expression, this reduction in CD38 expression was sustained out to 72 h and did not re-normalize. HLA-DR expression—also a marker of late cellular activation—similarly decreased with MSA stimulation out to 72 h (*P* = 0.008), though to a less pronounced magnitude (Fig. 3C, middle). Thus, MSA stimulation led to potent upregulation of HIV mRNA comparable to a maximal PMA/PHA stimulus, but without the concomitant global T-cell activation achieved with the latter compounds.

### MSA does not induce cellular proliferation and can inhibit mitogenic stimuli

In addition to MSA’s described effects on cell-autonomous circadian machinery, it has been shown to inhibit cell proliferation and induce apoptosis by promoting the nuclear localization of Forkhead box O (FOXO) transcription factors via phosphorylation (37). As cellular proliferation can also contribute to HIV reservoir persistence *in vivo*, we tested whether MSA’s cellular activation results in CD4+ T-cell proliferation as well.

**Fig 4:**
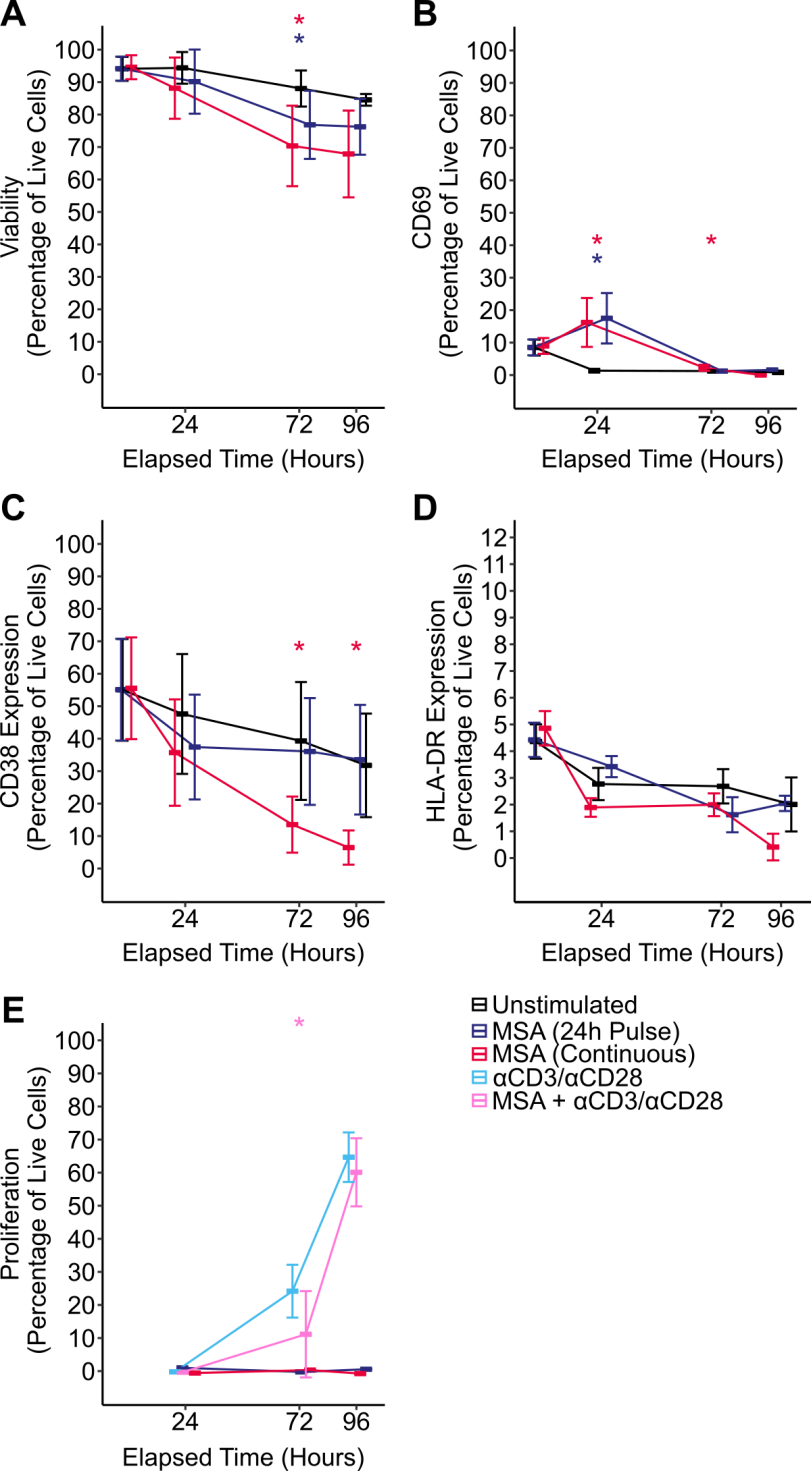
Continuous MSA stimulation does not induce profound cellular activation or proliferation and inhibits mitogen-induced proliferation. CD4+ T cells were isolated from PBMCs from HIV-1-negative donors cultured in the presence of 10 μM MSA for 24 h prior to drug wash off (dark blue) or for 96 h (red). After 24 h of MSA stimulation, cells were co-stimulated with αCD3/αCD28 Dynabeads for a further 72 h (pink). Cells not pre-treated with MSA prior to αCD3/αCD28 stimulation (light blue) were included as controls. Viability (**A**), CD69 (**B**), CD38 (**C**), and HLA-DR (**D**) expression were assessed by flow cytometry. Cellular proliferation (**E**) was assessed by dilution of CellTrace Violet, measured by flow cytometry. *n* = 4 independent donors, lines represent the mean ± SD. Statistical significance was determined using paired t-tests comparing to matched unstimulated timepoints; *P* < 0.05, *; *P* < 0.01.

Long-term culture of CD4+ T cells with MSA led to moderate loss in viability at 72 h; however, by 96 h, viability was not statistically different from unstimulated cells (Fig. 4A). Despite continuous culturing with MSA, CD69 expression was still self-limiting with a peak at 24 h before returning to baseline by 72 h (Fig. 4B); however, a sustained reduction in CD38 expression was observed (Fig. 4C). No change in HLA-DR expression was seen (Fig. 4D). Promisingly, neither a 24-h pulse nor 96-h continuous culture of CD4+ T cells from HIV-negative participants with MSA induced any CD4+ proliferation by measuring CellTrace dye dilution (Fig. 4E). Since MSA itself did not induce proliferation, we next tested whether it could inhibit cellular proliferation from additional mitogens. CD4+ T cells from HIV-negative participants were pre-treated with 10 μM MSA for 24 h before stimulation of the T-cell receptor (TCR) with anti-CD3/CD28 antibodies for a further 72 h (Fig. 4E). As expected, αCD3/CD28 stimulation alone caused significant CD4+ T-cell proliferation. Pre-treating cells with MSA, however, inhibited αCD3/CD28-induced cellular proliferation at 72 h (11.2% ± 6.5% proliferation cf. 25.1% ± 4.0% in those not pre-treated, *P* = 0.020), though this effect was lost by 96 h (*P* = 0.56). Thus, MSA itself is not mitogenic and is capable of delaying cellular proliferation in response to strong mitogenic stimuli through the TCR.

### MSA alters transcriptional profiles of CD4+ T cells to inhibit cellular activation and proliferation and activate cell stress pathways

**Fig 5:**
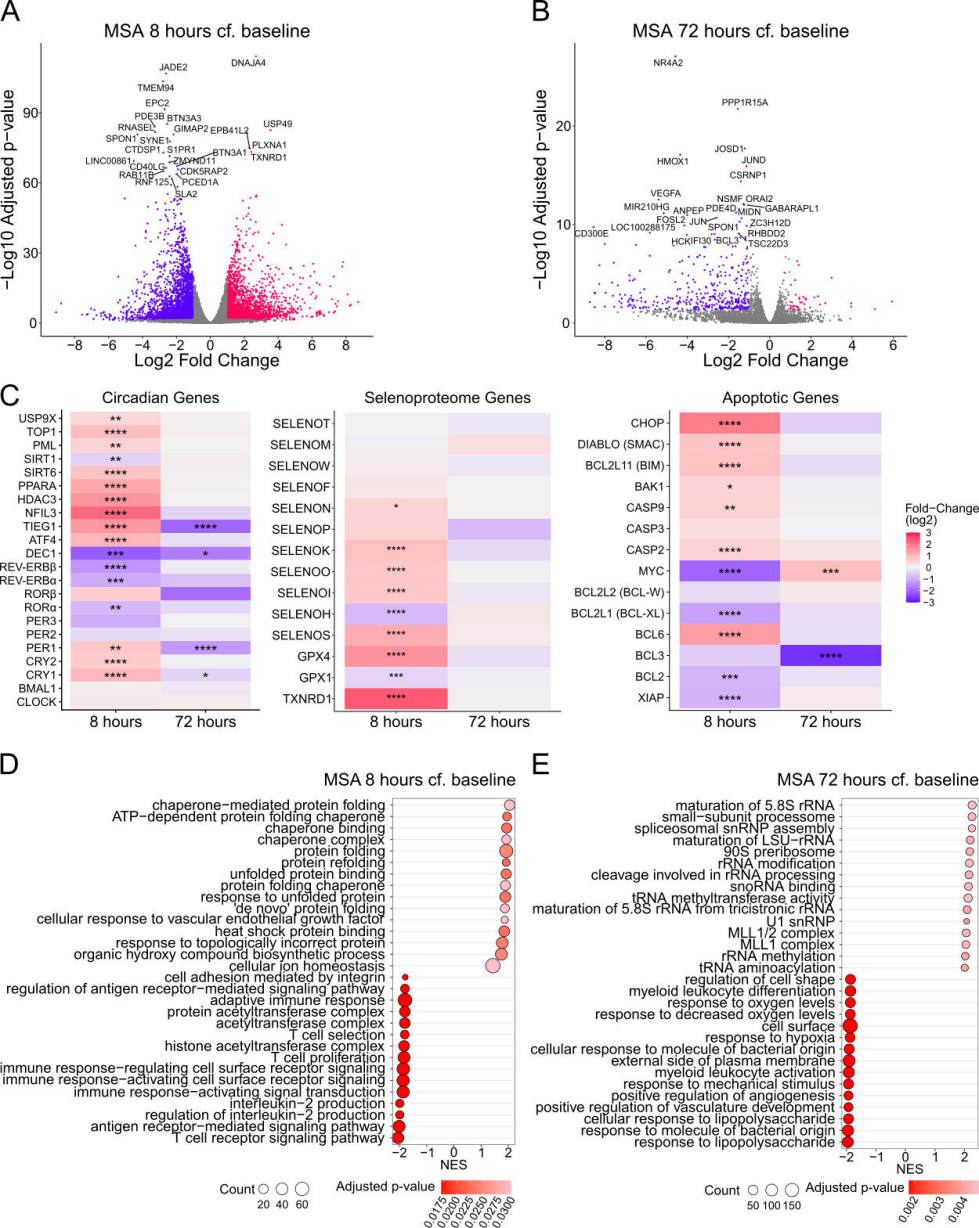
Bulk RNAseq identifies MSA-induced transcriptional changes in primary CD4+ T cells from PWH. Volcano plots display the significantly (FDR <0.05, |log_2_fold-change|>1) upregulated (pink) and downregulated (blue) genes 8 h (**A**) and 72 h (**B**) after MSA stimulation, compared to (cf.) baseline 0 h. (**C**) Transcriptional changes in the circadian genes (left), selenoproteome genes (middle), and apoptotic genes (right) induced by MSA stimulation. False-discovery rate <0.05, *; <0.01, **; <0.001, ***; <0.0001, ****. (**D and E**) Top 15 upregulated and down-regulated GO pathways by normalized enrichment score (NES) 8 and 72 h post-MSA stimulation, respectively.

Considering that 10% of the human genome is under circadian control and non-circadian effects of MSA administration have been described *in vitro*, we next examined the broader effects of MSA on the host transcriptome. CD4+ T cells from two PWH on ART for whom we had sufficient sample availability were cultured with MSA for 24 h, and bulk RNA sequencing was performed to identify the pleiotropic transcriptional effects of MSA at an early (8 h) and late (72 h, 48 h post-drug washout) timepoint. Compared to baseline 0 h, there were 4,817 (2,256 upregulated, 2,561 downregulated) and 443 (40 upregulated, 403 downregulated) differentially expressed genes at the 8 h and 72 h timepoints, respectively (gene expression >1 log_2_fold-change, adjusted *P*-value <0.05) (Fig. 5A and B).

Numerous genes involved in the regulation of cell-autonomous circadian clocks (*PER1*, *CRY1/2*, *DEC1*, *RORα, REV-ERBα/β*, *HDAC3, PPARA, SIRT1, SIRT6, PML, TOP1, USP9X,* and *TIEG1*) as well as clock-controlled genes important in immune function (*ATF4* and *NFIL3*) were perturbed by MSA stimulation at 8 h (Fig. 5C, left) (32, 38–54). Among the top downregulated genes at 8 h was *S1PR1* (log_2_(−2.4) fold-change), a surface receptor involved in lymphocyte lymph-node homing under circadian control (55). Among the top upregulated genes at 8 h was *TXNRD1* (log_2_(2.4) fold-change), an NADPH-dependent reductase involved in redox balance of selenium-containing compounds (56). Indeed, gene expression of multiple members of the *SELENO* and *GPX1* and *GPX4* selenoproteome family known to be altered by selenium uptake (57–59) was upregulated shortly after MSA stimulation (Fig. 5C, middle), confirming MSA’s incorporation by CD4+ T cells. Consistent with this, we observed an increase in selenium concentration within cells treated with MSA by inductively coupled plasma mass spectrometry (ICP-MS) (Fig. S7). These genes returned to baseline expression by 72 h, confirming the rapid kinetics of MSA’s stimulus of CD4+ T cells, similar to the observed cell-surface activation marker kinetics. Additionally, 8 h after MSA stimulation, gene expression of numerous anti-apoptotic genes (*BCL2, BCL3, BCL-XL, XIAP,* and *MYC*) was significantly downregulated, while pro-apoptotic genes (*CASP2, CASP9*, *BAK1, BIM,* and *SMAC*) were upregulated (Fig. 5C, right). Most of these genes returned to baseline expression by 72 h, except *MYC* (log_2_0.9 fold-change) and *BCL3* (log_2_(−2.67) fold-change), which were upregulated and downregulated, respectively.

An unbiased gene set enrichment analysis (GSEA) was used to identify transcriptional pathways that are dysregulated by MSA administration using Gene Ontology (GO) data sets. At 8 h post-stimulation, the top upregulated GO pathways were involved in protein folding/misfolding and heat shock protein binding (Fig. 5D), in line with the endoplasmic reticulum (ER) stress induced by selenocysteine incorporation into selenoproteins. The most downregulated GO pathways at 8 h were involved in T-cell activation via the TCR, IL-2 production, and T-cell proliferation, reflecting the decrease in late cellular activation marker expression on CD4+ T cells observed *in vitro,* as well as MSA’s inhibition of cellular proliferation in response to mitogens. Interestingly, *CD38* expression was not significantly altered (FDR = 0.32), indicating that its downregulation is likely post-transcriptional. Analysis of the linked genes among key enriched pathways (Fig. S8A) demonstrated a shared involvement of genes such as *CD3, CD4, CD28, FOXP3, PDE4B, PDE4D, STAT5B, CTLA4,* and *LCK* in the downregulation of GO pathways involved in TCR signaling and IL-2 production. Conversely, upregulated GO pathways involved in protein folding shared genes in the heat-shock protein (*HSP, DNAJ*) and chaperonins (*CCT*) (Fig. S8B), further confirming the effects of MSA on inducing ER stress, possibly due to protein misfolding initiating an unfolded protein response (UPR).

GSEA of MSA-stimulated cells at 72 h (48 h post-drug washout) revealed that most significantly downregulated GO pathways were involved in lymphocyte activation, angiogenesis, and responses to lipopolysaccharide/bacteria (Fig. 5E). Shared genes among these pathways included toll-like receptor (*TLR*) genes, interleukin (*IL*) genes, chemokine signaling gene families (*CXC, CCL*), caspases (*CASP*), VEGF/MAPK signaling, FOS/JUN signaling, and NF-kB signaling (Fig. S9A). The most significantly upregulated GO pathways were involved in ribosomal RNA processing, with shared genes belonging to the exosome complex (*EXOSC*) involved in RNA degradation (Fig. S9B). Together, these transcriptional changes after drug washout confirm inhibition of T-cell activation by MSA, and the upregulation of RNA processing/degradation may explain the loss of CA-US HIV RNA after 72 h without any expression of MS HIV RNA or virion release.

**Fig 6:**
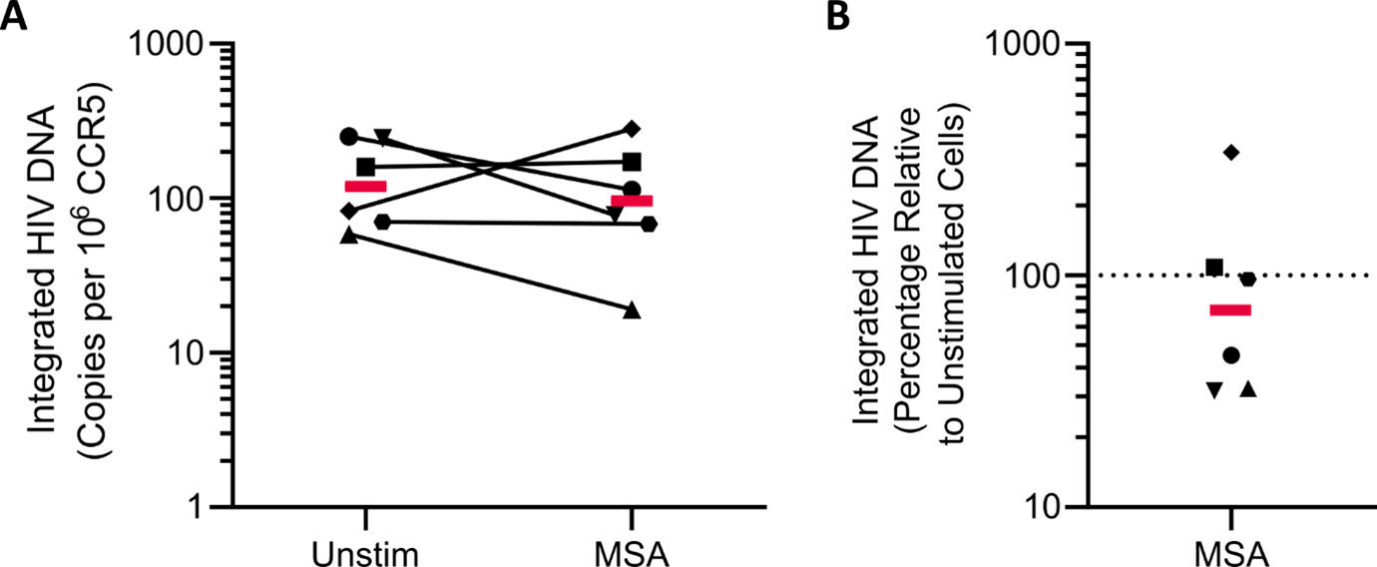
MSA does not significantly reduce integrated HIV-1 DNA levels. CD4+ T cells isolated from PWH were stimulated with 10 μM MSA for 24 h prior to drug washoff and further culture for 72 h, at which point integrated HIV-1 DNA was quantified by qPCR from cellular DNA and normalized to cell input (**A**). (**B**) Changes in integrated HIV-1 DNA were calculated as percentages relative to unstimulated cells cultured for 96 h. *n* = 6 independent donors, each symbol represents each individual participant, and red lines represent the median. Statistical significance was determined on log-transformed data compared to unstimulated cells using the Wilcoxon matched-pairs signed rank test.

Given MSA’s early induction of ER stress and potential later pro-apoptotic reprogramming of CD4+ T cells from PWH, we next assessed whether MSA could selectively deplete the HIV reservoir. After 72-h continuous MSA stimulation of CD4+ T cells *ex vivo,* integrated HIV DNA was not significantly altered (median 29% decline, *P* = 0.69) (Fig. 6).

**Fig 7:**
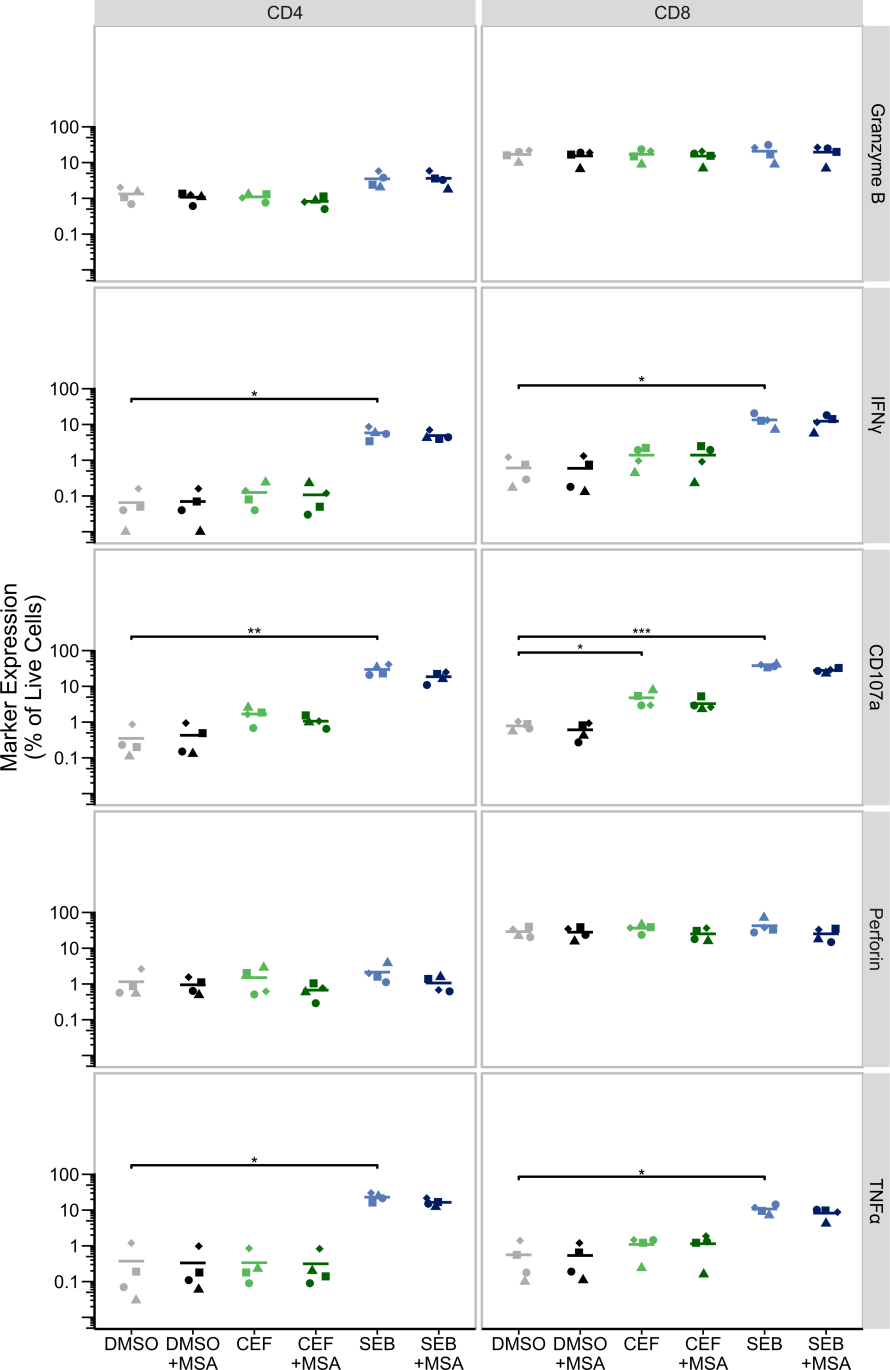
MSA does not affect antigen-mediated T-cell cytotoxicity. PBMC from HIV− donors were cultured in the absence/presence of MSA for 24 h prior to 6 h-stimulation with antigen pools of CEF (green), SEB (blue), or dimethyl sulfoxide (DMSO, black). CD4+ (left) and CD8+ (right) cytotoxicity were measured by degranulation marker expression. *n* = 4 independent donors, each symbol represents each individual participant, and horizontal lines represent the mean. Statistical significance was determined on log-transformed data using paired t-tests with Benjamini-Hochberg correction for multiple comparisons; *P* < 0.05, *; *P* < 0.01, **; *P* < 0.001, ***. IFNγ, interferon gamma; TNFα, tumor necrosis factor alpha.

As bulk RNAseq demonstrated that MSA induced transcriptional inhibition of CD4+ T-cell activation in monoculture, we next tested whether MSA inhibits T-cell cytotoxicity in response to antigen. Compared to DMSO alone, stimulation of PBMCs with antigen pools of CMV, EBV, and influenza (CEF) or staphylococcus enterotoxin B (SEB) significantly increased expression in CD8+ T cells of interferon gamma with SEB (IFNγ, *P* = 0.018), CD107a with SEB and CEF (*P* < 0.001 and *P* = 0.028, respectively), and tumor necrosis factor alpha with SEB (TNFα, *P* = 0.034) (Fig. 7, right); and in CD4+ T cells with SEB only, for IFNγ (*P* = 0.011), CD107a (*P* = 0.005), and TNFα (*P* = 0.028) (Fig. 7, left). However, co-culture of cells with MSA did not significantly decrease the expression of these cytokines or cytotoxic proteins in response to either CEF or SEB antigen (*P* > 0.05 for all markers comparing antigen with MSA to without MSA, Fig. 7).

## DISCUSSION

We have identified an organic selenium compound, MSA, that reactivates HIV latency alongside activation of the cell-autonomous circadian cycle of CD4+ T cells. MSA induced CA-US HIV RNA in latently infected cell lines and primary CD4+ T cells isolated from peripheral blood of PWH. In cell lines, this also resulted in sustained viral protein expression however, virion release was unchanged after MSA stimulation of primary CD4+ T cells, consistent with additional blocks to latency reversal in primary cells (60, 61). In both cell lines and primary cells, upregulation of the major circadian transcription factor, *BMAL1*, occurred rapidly after MSA stimulation, confirming MSA’s action to induce *BMAL1* transcription (34). In addition, we demonstrated a potent and sustained downregulation of CD38 expression on CD4+ T cells *ex vivo* and inhibition of proliferation induced by a strong mitogen, αCD3/CD28 antibody stimulus. These data clearly demonstrate the important role that BMAL1 plays in increasing HIV mRNA and are consistent with our observations that HIV transcription has a circadian rhythm *in vivo* in virally suppressed PWH, and CLOCK:BMAL1 can upregulate HIV transcription *in vitro* in an LTR-dependent manner (27, 28, 33). Our findings are consistent with other groups who have shown that pharmacologic inhibition of *BMAL1 in vitro* dampens HIV latency reversal (29, 62).

Using bulk RNAseq of MSA-treated CD4+ T cells from PWH, we showed perturbations of numerous circadian regulatory genes and clock-controlled transcription factors. UPR-specific apoptotic transcriptional changes demonstrated increased *CHOP* and decreased *BCL2* expression, as well as the upregulation of other pro-apoptotic genes (*CASP, BIM,* and *SMAC*) and downregulation of anti-apoptotic genes (*XIAP*, *BCL3, BCL-W,* and *BCL-XL*), many of which have been implicated in maintaining HIV persistence (13, 63–70). Together, these data demonstrate that MSA can increase HIV RNA levels, induce ER stress, and potentially prime cells for apoptosis as shown by bulk RNA sequencing. However, we did not demonstrate a statistically significant reduction in the reservoir *ex vivo* following MSA. Future work should focus on assessing more potent LRAs, together with MSA, as one strategy to enhance viral cytopathic effects (71–74). Furthermore, the inhibition of TCR-mediated CD4+ T-cell proliferation by MSA could prevent antigen-mediated clonal expansion of the HIV reservoir that may occur in the context of latency reversal, whereby HIV-infected HIV-specific CD4+ T cells are stimulated to proliferate and paradoxically expand the reservoir (75, 76). Moreover, expression of cytokines or cytotoxic proteins upon antigen exposure *in vitro* was not affected by MSA, indicating that MSA did not change T-cell functionality despite inhibiting CD4+ T cell TCR-mediated proliferation. Interestingly, MSA has been shown by others to increase MHC-I expression and decrease PDL1 and VEGF, allowing effective killing of cancer cells by CD8 T cells (77, 78).

MSA also led to profound downregulation of CD38 expression on CD4+ T cells from PWH. Others have demonstrated that CD38 downregulation reduces HIV-specific cytokine production in CD4+ and CD8+ T cells from PWH (79). CD38 is an ectoenzyme playing a major role in nicotinamide adenine dinucleotide (NAD) catalysis, and CD38 downregulation has been shown to improve mitochondrial function (79, 80). Indeed, it has recently been demonstrated in a mouse model of Alzheimer’s disease that inhibition of REV-ERBα—which negatively controls BMAL1 expression—decreases CD38 expression to regulate brain NAD+ levels and restore astrocyte function to protect against neurodegeneration (81). While this may also be beneficial to reduce chronic immune senescence and aging caused by long-term HIV infection, the effects of enhanced mitochondrial function on latency reversal, death of the infected cell, or HIV-specific CD8+ T cells remain unknown and warrant further exploration. In addition, combination strategies of MSA together with other LRAs or pro-apoptotic compounds such as SMAC-mimetics or BCL2 inhibitors may result in an increased reservoir clearance (13, 82, 83).

One attractive feature of organic selenium compounds is their safety profiles being widely tested in clinical trials. Selenium supplementation is safe in PWH off ART, as has high-dose repeated selenium supplementation in selenium-replete HIV-negative males (84, 85). Although some cellular toxicity was observed *in vitro* with MSA in this study, selenium bioavailability *in vivo* is improved when MSC is administered as opposed to MSA. MSC is metabolized into methylselenol (for selenoprotein incorporation) by β-lyases, which occurs at a slower rate than the nonenzymatic metabolism of MSA (86) and would thus likely reduce the toxicity of selenium supplementation *in vivo*. Nonetheless, a single-dose *in vivo* supplementation with MSC showed that selenium plasma concentrations reached their maximum within a few hours before returning to baseline within ~24 h, likely reflecting MSC’s rapid metabolism and incorporation into tissues as well as its clearance *via* the urine and breath (86, 87). Indeed, we demonstrated that within just 8 h of MSA stimulation, cells had already increased intracellular selenium concentrations, which rapidly—but transiently—affected the transcriptional response to selenium incorporation into proteins.

A limitation of this study was that expression profiles of HIV and circadian proteins, or cytokines, were not measured to assess whether they tracked with the observed changes in RNA levels or impacted immune recognition by antibody binding. Additionally, although we and others have demonstrated that *BMAL1* upregulation can induce HIV transcription in an LTR-mediated manner (28, 33), we did not directly demonstrate a direct link between MSA-induced *BMAL1* expression and HIV transcription.

Our proposed model is that MSA induces BMAL1 expression, which, upon translation and heterodimerization with CLOCK, binds to an E-box motif present in the HIV LTR promoter, essentially enacting circadian transcriptional regulation on the HIV provirus as though it were any other clock-controlled gene. In our study, the effects of MSA on latency reversal were short-lived, with CA-US HIV RNA being upregulated for only 24 h prior to returning to baseline. These kinetics may be due to drug washout after 24 h, the rapid metabolism of MSA in culture, or the homeostatic response imparted by the cell-autonomous circadian cycle, evidenced by increased gene expression of the circadian repressor, *PER*, in primary cells that occurred after *BMAL1* induction. Additionally, while not examined in this study, the synchronization of cell-autonomous circadian machinery *in vitro,* such as using the glucocorticoid dexamethasone (88, 89), may have better highlighted MSA’s effects on circadian and HIV transcription; however, this synchronization itself would have also impacted HIV transcription as the LTR contains glucocorticoid response elements (90, 91).

Alternatively, Hu et al. demonstrated that TGFβ inducible early gene-1 (TIEG-1)—a zinc finger Krüppellike transcription factor—repressed *BMAL1* transcription likely by binding to Sp1-binding sites within the gene’s promoter to repress the activating Sp1 activity (34). They thus proposed that *BMAL1* induction by MSA was mediated by the removal of the repressive TIEG-1 on the gene promoter. HIV-1 similarly contains three Sp1-binding sites which affect basal and tat-induced transcriptional activity (92, 93), and TIEG-1—as well as TRIM22—has been shown to inhibit HIV-1 transcription *in vitro* by inhibiting Sp1 interaction (94, 95). Thus, the coincident peaks in *BMAL1* and CA-US HIV RNA by MSA treatment may be explained by an inhibition of TIEG-1, resulting in increased HIV-1 transcription through increased Sp1 binding. Another possibility could be that HIV reactivation was induced by MSA as a consequence of the UPR and ER-stress response. Activating transcription factor 4 (ATF4) is activated as part of the UPR and has been shown to reactivate latency (96, 97). However, there is a strong interaction between circadian rhythms and cell stress responses (74, 98).

In addition to a direct effect of circadian transcription factors, circadian rhythms of T cells and their immune function are strongly linked: lymphocyte trafficking, cytokine production, proliferation, antigen processing, and vaccination responses show strong circadian rhythmicity (55, 99–103). Furthermore, *BMAL1* expression has been linked to lymphocyte activation and exhaustion and responsiveness to immune checkpoint blockade (104, 105). Notably, the circadian clock governs T_H_17 differentiation via regulation of the orphan nuclear receptor, RORγt, which affects susceptibility to inflammatory disease (44). Despite not having demonstrated causality, the fold-change in *BMAL1* RNA following MSA was strongly positively correlated with the fold-change of *PER1* and unspliced HIV RNA. Furthermore, numerous other genes, such as the *CXCL* family, *S1PR1*, and *IDO1*, have been described to be under circadian influence and were among the top differentially expressed genes after MSA treatment (55, 103). Furthermore, MSA potently upregulated RNA levels of the deubiquitination enzyme, *USP49*, which has been linked to the modulation of circadian rhythms *in vivo,* and also stabilizes apolipoprotein B mRNA editing enzyme catalytic polypeptide-like 3G (APOBEC3G) expression to enhance its inhibition of HIV replication *in vitro* (106–108). This indicates a likely circadian component to MSA’s immunomodulatory effects on viral reactivation and cellular processes.

In conclusion, the selenium derivative MSA can increase BMAL1 and HIV RNA expression in HIV-infected cells that persist in PWH on ART, as well as potentially preventing proliferation of infected cells and possibly sensitizing infected cells to apoptosis. Further research is needed to assess whether MSA, together with LRAs that target different pathways, or pro-apoptotic drugs may enhance the death of latently infected cells *ex vivo*.

## MATERIALS AND METHODS

### Circadian-modulating compounds

MSC (Abcam, Cambridge, United Kingdom) and MSA (Sigma Aldrich) were obtained as powdered drugs and reconstituted in RNAse-free, DNAse-free H_2_O (Life Technologies, Waltham, MA) at stock concentrations of 100 mM. Nobiletin and ivermectin were obtained as powdered drugs (Sigma Aldrich, Burlington, MA) and reconstituted in DMSO (Sigma Aldrich) at stock concentrations of 20 mM and 50 mM, respectively. Resveratrol was obtained as a powdered drug (Sigma Aldrich) and reconstituted in absolute ethanol (VWR International, Radnor, PA) to stock concentrations of 200 mM. All reconstituted drugs were aliquoted into single-use aliquots and stored below −20°C until the day of use.

### Drug screening of latency reversal by circadian-modulating compounds in latently infected cell lines

Suspension cell lines, Jurkat and J-Lat Tat-IRES-GFP clone A2 (NIH AIDS Reference Reagent Program, [355]), were cultured in Roswell Park Memorial Institute 1640 (RPMI) media supplemented with 10% heat-inactivated fetal bovine serum (FBS) and 100 units/mL penicillin, 100 μg/mL streptomycin, and 292 μg/mL L-glutamine (PSG) (RF10). 2 × 10^5^ viable cells were plated into each well of a 96-well U-bottom plate in 100 μL RF10, and drug titrations were generated by serial dilution of each compound in their respective diluent. Cells were cultured with the respective compounds for 24 h, after which plates were pelleted by centrifugation at 300 × *g* for 5 min at room temperature and washed twice in fresh RF10 by vacuum aspiration of supernatant to remove any residual drug. Cells were then resuspended in fresh RF10 and cultured for a further 24 h (48-h total culture period).

At 24 h and 48 h post-stimulation, cultures were harvested in triplicate for assessment of viability and viral reactivation. Plates were pelleted by centrifugation at 300 × *g* for 5 min at room temperature and washed twice in FACS-wash buffer (phosphate-buffered saline [PBS] supplemented with 10% FBS and 1 μM ethylenediaminetetraacetic acid [EDTA]) before being stained with 100 μL of 1/12,500 Live/Dead Fixable Cell Death Stain Aqua or Violet (ThermoFisher Scientific, Waltham, MA) for 20 min at room temperature away from light. Residual stain was then removed by washing cells twice by centrifugation and resuspension of cells in FACS-wash buffer before fixing in 1% paraformaldehyde in PBS.

Flow cytometry was performed on the LSR Fortessa (BD Biosciences, Franklin Lakes, NJ) using the high-throughput system coupler. Viability was measured by the detection of Live/Dead Fixable Cell Death Aqua or Violet stain using the bandpass filter 525/50 or 450/50 Violet, respectively, and GFP expression detected using the 530/30 Blue bandpass filters. Forward scatter (FSC) and side scatter (SSC) height (H), width (W), and area (A) were measured to allow for cell discrimination based on size and granularity (FSC-A vs SSC-A), as well as discrimination between singlet cells and doublets/triplets (FSC-H vs FSC-A). The representative gating strategy is depicted in Fig. S1A. Results were analyzed using FCS Express v7 (*De Novo* Software, Thornhill, Ontario, Canada), with autocompensation performed using single-stain controls and gating set using fluorescence minus one controls.

### MSA stimulation of latently infected cell lines

10^6^ viable J-Lat 10.6 and ACH2 cells (NIH AIDS Reference Reagent Program [109, 110]) were cultured in RF10 at a density of 2 × 10^6^ cells/mL and stimulated with 10 μM MSA (Sigma Aldrich). As positive controls, J-Lat 10.6 cells were stimulated with 16 nM PMA (Sigma Aldrich) and 500 nM ionomycin (Sigma Aldrich), and ACH2 cells were stimulated with 10 nM PMA. After 18 h of culture at 37°C, 5% CO_2_, the drug was washed out by centrifugation and resuspended cells in fresh media.

At 8, 24, and 48 h post-stimulation, cultures were harvested in duplicate for assessment of viral reactivation and cell viability. 5 × 10^5^ cells were pelleted, resuspended, and washed twice in FACS-wash buffer and stained with 100 μL of 1/2,000 Live/Dead Fixable Cell Death Stain Violet (ThermoFisher Scientific) for 20 min at room temperature. Cells were washed twice in FACS-wash buffer, and J-Lat 10.6 cells were resuspended in 1% paraformaldehyde in PBS.

After being stained with Live/Dead Violet, ACH2 cells were then stained for intracellular p24 (HIV-1 Gag) expression. Cells were pelleted and resuspended in 100 μL cold BD Cytofix/Cytoperm fixation/permeabilization solution (BD Biosciences) and incubated at 4°C for 20 min. After permeabilization, samples were pelleted by centrifugation and washed twice in BD Perm/Wash Buffer diluted 1/10 in ultrapure H_2_O. Samples were then incubated with 50 μL 1/1,000 anti-p24 PE KC57-RD1 (Cat # 6604667, Beckman Coulter, Brea, CA) or 1/50 mouse IgG1 PE isotype control (Cat# 555749, BD Biosciences) at 4°C for 30 min and washed twice in Perm/Wash buffer. Cell viability and HIV protein expression were assessed by flow cytometry, measuring cell viability by Live/Dead Violet staining and HIV expression measured as GFP expression and p24-PE staining in J-Lat 10.6 and ACH2 cells, respectively. The representative gating strategy is displayed in Fig. S2.

### MSA stimulation of primary peripheral blood CD4+ T cells

PBMCs were isolated by Ficoll-Paque (Amersham Pharmacia Biosciences AB, Sweden) as previously described (111) from leukaphereses of virally suppressed PWH (ethics approval—including informed consent from participants—obtained from Alfred Hospital, Melbourne, Australia ID 1545227, 1448, and 48-16, and the University of Melbourne, Melbourne, Australia ID 1443162) (Table 1). CD4+ T cells were isolated by negative magnetic separation using the EasySep Human CD4+ T cell Isolation Kit (StemCell Technologies, Vancouver, Canada) as per the manufacturer’s guidelines. The purified CD4+ T cells were resuspended in RF10 to a density of 2 × 10^6^ cells/mL and supplemented with 1 U/mL human IL-2 (Sigma Aldrich) and 1 μM raltegravir (Jomar Life Research, Victoria, Australia). After an overnight rest, 5 × 10^6^ cells, at a density of 2.5 × 10^6^ cells/mL, were stimulated with 10 μM MSA, or 10 nM PMA and 10 μg/mL PHA (Thermo Fisher Scientific) as a positive control. After 24 h, the drug was washed out by centrifugation and resuspending cells in fresh media supplemented with 1 U/mL IL-2 and 1 μM raltegravir.

**TABLE 1:** Leukapheresis cohort participant demographics^*a*^

Participant	Age (years)	CD4+ count (cells/μL)	CD4+ nadir (cells/μL)	ART regimen
LKA003	48	662	117	EFV/TDF/FTC
LKA012	56	744	624	RAL/DRV
LKA013	58	520	27	N/A
LKA015	42	681	198	NVP, TDF/FTC
LKA016	67	534	315	ABC/3TC/DTG
LKA019	28	1,072	732	TAF/FTC/RPV
PRA001	64	403	10	ATV, TDF/FTC
PRA002	48	1,460	698	ABC/3TC, EFV
PRA009	49	474	42	EVG/TAF/FTC/c
USCF3162	56	586	200	DRV, RTV, ABC/DTG/3TC
Cohort median	52.5	624	199	

^*a*^ 3TC, lamivudine; ABC, abacavir; ATV, atazanavir; /c, cobicistat; DRV, darunvair; DTG, dolutegravir; EFV, efavirenz; FTC, emtricitabine; N/A, not available; NVP, nevirapine; RAL, raltegravir; RPV, rilpivirine; TAF, tenofovir alafenamide; TDF, tenofovir disoproxil fumarate.

At 8, 24, and 72 h post-stimulation, ~7.5 × 10^5^ cells were harvested in duplicate for measurement of surface activation marker expression and viability by flow cytometry. Remaining cells were pelleted, supernatant stored for HIV-1 RNA quantification, and cell pellets lysed in Trizol reagent (ThermoFisher Scientific). Cells were washed twice in FACS-wash buffer and stained with 10 µL 1/125 Live/Dead Fixable Cell Death Stain Violet and incubated at room temperature for 20 min. After incubation, cells were stained with 2 μL each of mouse anti-human CD3 BV711 (Clone UCHT1, Cat#563725, BD Biosciences), CD4 PE-Texas Red (Clone S3.5, Cat#MHCD0417, ThermoFisher Scientific), CD38 PE (Clone HB7, Cat#347687, BD Biosciences), CD69 AlexaFluor 647 (Clone FN50, Cat#310918, Biolegend, San Diego, CA), and 5 μL HLA-DR FITC (Clone L243, Cat#347363, BD Biosciences) without removal of Live/Dead Violet stain. Cells were incubated with the fluorescently labeled antibodies for 20 min at room temperature, after which cells were washed twice in FACS-wash buffer and fixed in 1% paraformaldehyde in PBS. The representative gating strategy is displayed in Fig. S4.

### Quantification of HIV-1 RNA and circadian gene expression by qPCR

RNA was isolated from Trizol reagent (ThermoFisher Scientific) as per the manufacturer’s guidelines. cDNA was synthesized as previously described (28), using 500 ng RNA input per 20 μL reaction, with random hexamers (75 ng/μL Life Technologies), oligo(d)T (12.5 ng/μL, Life Technologies), dNTP (50 nM, Promega, Madison, WI), DTT (5% [vol/vol], Life Technologies), first strand buffer (20% [vol/vol], Life Technologies), RNase Out (2.5% [vol/vol], Life Technologies), and Superscript III reverse transcriptase (5% [vol/vol], Life Technologies). RT-qPCR was performed to quantify the circadian genes, *CLOCK, BMAL1, PER1-3, CRY1-2,* and the housekeeping genes, *GAPDH* and *B2M*, for cell lines and primary cells, respectively, using the ∆∆Ct relative quantification method as previously described (28). Housekeeping genes were confirmed to remain within 0.5- to 2-fold of baseline over 48–72 h of MSA stimulation, confirming our ability to measure changes in circadian gene expression <0.5-fold or >2-fold (Fig. S10).

CA-US) and MS HIV-1 RNA was quantified as previously described (7, 35). For cell lines, the pre-amplification step was not performed due to the high copy number of transcripts in the latently infected cell lines. Samples were run in two to four replicates, and HIV RNA expression was normalized to RNA input.

### Quantification of supernatant HIV-1 RNA

HIV-1 RNA was quantified in cell culture supernatants by the Victorian Infectious Diseases Reference Laboratory (VIDRL, Melbourne, Victoria) using the Alinity m (Abbott Molecular, Des Plaines, IL) with a limit of detection (LOD) of 20 copies/mL and dynamic range of 10-2 × 10^6^ copies/mL. Values were expressed as copies per mL of input, and samples below the LOD were assigned as 20 copies/mL.

### Quantification of integrated HIV-1 DNA by qPCR

In integrated HIV-1 DNA experiments, total CD4+ T cells were isolated from PWH on ART and stimulated with MSA as described above, with 2 × 10^6^ in 1 mL instead of 5 × 10^6^ cells in 2 mL. After a 24-h stimulation with 10 μM MSA and subsequent drug washout, cells were cultured for an additional 72 h, i.e. a 96-h total culture period, after which cells were pelleted, cell pellets frozen at −80°C and lysed in 10 mM Tris HCl (pH 8), 1 mM EDTA, and 0.002% Triton X-100/SDS in distilled H_2_O supplemented with 0.8 mg/mL Proteinase K. Lysates were then homogenized by incubating at 56°C for 60 min and then 95°C for 10 min to inactivate the proteinase K and then chilled to 4°C and stored at −80°C until further use. A nested Taqman-probe qPCR was performed on 5 μL lysate to measure integrated HIV-1 DNA and normalized to sample input (by CCR5 copy number using a SYBR Brilliant III- [Agilent Technologies, Santa Clara, CA] based qPCR assay), as previously described (112). The number of cell equivalents of DNA assayed was between 1.14 × 10^4^and 1.72 × 10^5^ cells per replicate.

### Assessment of cell proliferation after MSA stimulation of CD4+ T cells from HIV− participants

PBMCs were isolated from buffy coats from HIV-1-negative donors from the Australian Red Cross Lifeblood (ethics approval from University of Melbourne ID 1433071 and 13194) by Ficoll-Paque. Total CD4+ T cells were isolated using the EasySep Human CD4+ T-cell Isolation Kit (StemCell Technologies) and rested overnight in RF10 + 1 U/mL IL-2 at a density of 2 × 10^6^ cells/mL. Cells were then resuspended at 2 × 10^7^ cells/mL in PBS + 0.1% bovine serum albumin (BSA, Sigma Aldrich) and stained with 5 μM CellTrace Violet (ThermoFisher Scientific) and incubated at 37°C for 20 min, with regular mixing. Labeling was then stopped by adding a 5× volume of cold PBS + 0.1% BSA, and cells were washed twice in PBS + 0.1% BSA. 5 × 10^5^ cells, at a density of 2 × 10^6^ cells/mL, were stimulated with 10 μM MSA for 24 h prior to being stimulated with αCD3/αCD28 human dynabeads (Life Technologies) at a density of three beads/cell. Cells were cultured for an additional 72 h (96-h total culture period) to allow for the proliferation of cells. At 24, 72, and 96 h post-MSA stimulation (i.e., 0, 48, and 72 h post-αCD3/αCD28 stimulation), cells were stained for viability and cell surface markers as above. Dynabeads were removed prior to flow staining by placing samples on a magnet for 2 min. Results were analyzed using FCS Express v7, with the proliferation curves fitted using the FCS Express proliferation tool, setting the “unproliferated” peak as the CellTrace Violet fluorescence of unstimulated cells at each timepoint. The representative gating strategy is displayed in Fig. S6.

### Bulk RNA sequencing of MSA-stimulated primary CD4+ T cells from PWH

Primary CD4+ T cells were isolated from PBMCs from two virally suppressed PWH and stimulated with 10 µM MSA for 24 h, as above, before washing out the drug and culturing for a further 48 h (total 72 h culture). At 8 h and 72 h, cells were lysed, and RNA was isolated as above. Bulk RNA sequencing was performed by the Australian Genome Research Facility (AGRF, Melbourne, Australia) with cDNA libraries generated following the manufacturer’s guidelines (Illumina TruSeq stranded RNA) and sequenced using the Illumina NovaSeq platform. The per-base sequence quality was >87% bases above Q30 across all samples. The reads were also screened for the presence of any Illumina adapter/overrepresented sequences and cross-species contamination. The cleaned sequence reads were then aligned against the Homo sapiens genome (Build version HG38). The STAR aligner (v2.5.3a) was used to map reads to the genomic sequences, and counts of reads mapping to each gene were summarized. Differential gene expression was performed using edgeR (https://bioconductor.org/packages/release/bioc/html/edgeR.html [113]), using the TMM normalization method to normalize counts between samples before comparing normalized gene counts at 8 and 72 h to baseline expression at 0 h. Genesets were annotated using the ensembldb v2.27.1 (114) (doi:10.18129/B9.bioc.ensembldb) and ranked as sign*-log_10_(p-value). GSEA of GO pathways was performed using clusterprofiler v4.11.0 (115) (doi: 10.18129/B9.bioc.clusterProfiler) with 10,000 permutations, minimum geneset size of 20 and maximum of 800, and Benjamini-Hochberg correction using R statistical software (2024-02-02 r85855 ucrt; R Core Team 2024). Gene concept networks of the top differentially expressed gene sets were created using enrichplot (v1.23.1; doi: 10.18129/B9.bioc.enrichplot).

### Selenium quantification by ICP-MS

Cell pellets containing approximately 2 × 10^6^ cells were lysed in 50 µL 65% nitric acid (HNO_3_) (Suprapure Nitric Acid Supelco). Samples were heated at 96°C for 30 min and allowed to cool. Samples were diluted with ultrapure water (18.2 MΩ; Merk Millipore, Australia) to 1% HNO_3_ final concentration. Samples were centrifuged at 15,000 × *g* for 20 min, and the supernatant was transferred to a new tube. Sample blanks were prepared identically to the samples for analysis. An Agilent 8900 triple quadrupole ICP-MS (Agilent Technologies) was tuned and optimized using a tuning solution containing 1 μg/L of cerium (Ce), cobalt (Co), lithium (Li), thallium (Tl), and Y in 2% (vol/vol) HNO_3_ (Agilent Technologies, Australia). The ion intensity at m/z 78 and 80 (selenium) was monitored in a hydrogen gas analysis mode. The instrument was calibrated using a 13-point calibration curve for iron using commercially available multi-element standards at 0, 0.01, 0.03, 0.1, 0.3, 0.5, 1, 2.5, 5, 10, 25, 50, and 100 parts per billion (ppb) in 3.5% HNO_3_ (Multi-Element Calibration Standard 2A, Agilent Technologies, USA). Yttrium (89Y) and Scandium (45Sc) (Agilent Technologies) were used as an internal reference elemental standard at a concentration of 0.1 μg/mL and used to normalize recovery across all samples. All samples, calibration standards, and internal standards were introduced to the nebulizer using a peristaltic pump and T-piece for sample mixing at a flow rate of 0.4 mL/min. The sample uptake time was 25 s, and the stabilization time was 15 s. The data were collected in spectrum mode with the average of three technical replicates, 50 sweeps, and three points across the peak. The ICP-MS operating parameters were established following the manufacturer’s guidelines, with further optimization tailored specifically for iron analysis in a batch-specific mode prior to each experiment. The instrument was operated in Single Quad scan type with an RF power of 1550 W and an RF matching voltage of 1.8 V. The nebulizer gas flow rate was maintained at 1.05 L/min, while the extraction voltages were set at −5 V for Extract 1 and −250 V for Extract 2. The Omega Bias and Omega Lens voltages were optimized to −150 V and 10 V, respectively, with the Deflect voltage adjusted to −13 V. Hydrogen gas was introduced at a flow rate of 4 mL/min to enhance sensitivity and reduce interference. The octopole bias was maintained at −18 V to ensure optimal performance during iron analysis. The LOD (lowest analyte concentration distinguishable from the blank) and limit of quantification (LOQ—lowest concentration quantifiable with acceptable accuracy and precision) were determined to evaluate the sensitivity and reliability of the analytical method. The calculations were performed using the Standard Deviation of Blank Method. For the sample blank, the LOD was calculated as 0.0106 µg/L, and the LOQ as 0.0356 µg/L. The blank equivalent concentration was 0.00899 µg/L, indicating that both the LOD and LOQ exceed the baseline signal of the blank. This ensures that the method is suitable for detecting and quantifying analyte concentrations above the baseline noise. Furthermore, all sample concentrations were significantly higher than both the LOD and LOQ, confirming the method’s sensitivity, accuracy, and precision for the data set.

### T-cell cytotoxic functionality assay

A TCR stimulation assay was performed to determine the effect of MSA on immune effector cells. PBMC from HIV− donors were treated with 5 µM MSA for 24 h, subsequently resuspended at 10^6^ cells in 100 μL and stimulated with either 2 µg/mL of SEB (Sigma-Aldrich); 1 µg/mL of a peptide pool of CEF antigens (NIH-ARP 9808); or DMSO (0.4%) as a vehicle control for a total of 6 h. GolgiStop (BD Biosciences) and Royal Yellow 610 Mouse anti-human CD107a antibody (Cat# 5042890 BD Biosciences) were added during the stimulation period. Cells were washed twice in FACS-wash buffer and stained with 1/88 LIVE/DEAD Fixable Blue for 20 min at room temperature. Cells were subsequently washed twice in FACS-wash buffer and stained with surface staining mix containing 5% Brillian Stain Buffer (Cat# 563794 BD Biosciences); APC mouse anti-human MHC-1 (Cat# 17998342 Invitrogen); Brilliant UV 496 Mouse anti-human CD4 (Cat# 612936 BD Biosciences); and Brilliant UV 805 mouse anti-human CD8 (Cat# 612889 BD Biosciences) in the dark for 30 min at room temperature. Following two washes in FACS-wash buffer, cells were permeabilized using Cytofix Cytoperm (Cat# 554714) for 30 min at 4°C. Cells were then stained with intracellular staining mix containing permeabilization buffer (10% Perm/Wash Buffer Cat#554723 BD Biosciences in dH_2_O); Pacific Blue mouse anti-human perforin (Cat# B436925 Biolegend); Brilliant Violet 750 mouse anti-human tumor necrosis factor alpha (Cat# 566359 BD Biosciences); Real Blue 705 mouse anti-human interferon gamma (Cat# 570247 BD Biosciences); Alexa Fluor 700 mouse anti-human granzyme B (Cat# 560213 BD Biosciences); and APC H7 mouse anti-human CD3 (Cat# 560176 BD Biosciences) for 30 min at 4°C. Following two washes with permeabilization buffer, cells were resuspended in 1% paraformaldehyde in PBS. Samples were acquired on a Cytek Aurora Flow Cytometer, processed by SpectroFlo software, and analyzed by FCS Express.

### Statistical analysis

Data were analyzed and figures were generated using GraphPad Prism 9.3.1 (Graphpad Software, Boston, MA) or R statistical software. For experiments with *n* < 6, statistical significance was determined using paired t-tests, assuming the data are normally distributed, to account for the limitations in statistical power for non-parametric data with small experiment numbers. For experiments with *n* ≥ 6, Wilcoxon matched-pairs signed rank tests were used to determine statistical significance. HIV RNA and DNA data were log-transformed prior to statistical testing. *P* < 0.05, *; *P* < 0.01, **; *P* < 0.001, ***; *P* < 0.0001, ****.

## Data Availability

Values for all data points in graphs are reported in Data Set S1. Raw bulk RNA sequencing reads are available upon request to the corresponding author, as our human participants consented to genetic testing but did not explicitly consent to these data being made accessible via online data repositories.

## SUPPLEMENTAL MATERIAL

Data Set S1 jvi.01983-25-s0001.xlsx Data values used in figures.

Supplemental material jvi.01983-25-s0002.docx Table S1; Fig. S1 to S10.

ASM does not own the copyrights to Supplemental Material that may be linked to, or accessed through, an article. The authors have granted ASM a non-exclusive, world-wide license to publish the Supplemental Material files. Please contact the corresponding author directly for reuse.

## References

[1] Bekker L-G, Beyrer C, Mgodi N, Lewin SR, Delany-Moretlwe S, Taiwo B, Masters MC, Lazarus JV. 2023. HIV infection. Nat Rev Dis Primers 9:42. doi:10.1038/s41572-023-00452-3 37591865

[2] Chun TW, Davey RT, Engel D, Lane HC, Fauci AS. 1999. Re-emergence of HIV after stopping therapy. Nature New Biol 401:874–875. doi:10.1038/44755 10553903

[3] Finzi D, Blankson J, Siliciano JD, Margolick JB, Chadwick K, Pierson T, Smith K, Lisziewicz J, Lori F, Flexner C, Quinn TC, Chaisson RE, Rosenberg E, Walker B, Gange S, Gallant J, Siliciano RF. 1999. Latent infection of CD4+ T cells provides a mechanism for lifelong persistence of HIV-1, even in patients on effective combination therapy. Nat Med 5:512–517. doi:10.1038/8394 10229227

[4] Ren Y, Huang SH, Patel S, Alberto WDC, Magat D, Ahimovic D, Macedo AB, Durga R, Chan D, Zale E, Mota TM, Truong R, Rohwetter T, McCann CD, Kovacs CM, Benko E, Wimpelberg A, Cannon C, Hardy WD, Bosque A, Bollard CM, Jones RB. 2020. BCL-2 antagonism sensitizes cytotoxic T cell–resistant HIV reservoirs to elimination ex vivo. J Clin Invest 130:2542–2559. doi:10.1172/JCI132374 32027622 PMC7191002

[5] Archin NM, Liberty AL, Kashuba AD, Choudhary SK, Kuruc JD, Crooks AM, Parker DC, Anderson EM, Kearney MF, Strain MC, Richman DD, Hudgens MG, Bosch RJ, Coffin JM, Eron JJ, Hazuda DJ, Margolis DM. 2012. Administration of vorinostat disrupts HIV-1 latency in patients on antiretroviral therapy. Nature 487:482–485. doi:10.1038/nature11286 22837004 PMC3704185

[6] Shan L, Deng K, Shroff NS, Durand CM, Rabi SA, Yang H-C, Zhang H, Margolick JB, Blankson JN, Siliciano RF. 2012. Stimulation of HIV-1-specific cytolytic T lymphocytes facilitates elimination of latent viral reservoir after virus reactivation. Immunity 36:491–501. doi:10.1016/j.immuni.2012.01.014 22406268 PMC3501645

[7] Elliott JH, McMahon JH, Chang CC, Lee SA, Hartogensis W, Bumpus N, Savic R, Roney J, Hoh R, Solomon A, Piatak M, Gorelick RJ, Lifson J, Bacchetti P, Deeks SG, Lewin SR. 2015. Short-term administration of disulfiram for reversal of latent HIV infection: a phase 2 dose-escalation study. Lancet HIV 2:e520–e529. doi:10.1016/S2352-3018(15)00226-X 26614966 PMC5108570

[8] Kwon KJ, Timmons AE, Sengupta S, Simonetti FR, Zhang H, Hoh R, Deeks SG, Siliciano JD, Siliciano RF. 2020. Different human resting memory CD4^+^ T cell subsets show similar low inducibility of latent HIV-1 proviruses. Sci Transl Med 12:eaax6795. doi:10.1126/scitranslmed.aax6795 31996465 PMC7875249

[9] Ho Y-C, Shan L, Hosmane NN, Wang J, Laskey SB, Rosenbloom DIS, Lai J, Blankson JN, Siliciano JD, Siliciano RF. 2013. Replication-competent noninduced proviruses in the latent reservoir increase barrier to HIV-1 cure. Cell 155:540–551. doi:10.1016/j.cell.2013.09.020 24243014 PMC3896327

[10] Pollack RA, Jones RB, Pertea M, Bruner KM, Martin AR, Thomas AS, Capoferri AA, Beg SA, Huang SH, Karandish S, Hao H, Halper-Stromberg E, Yong PC, Kovacs C, Benko E, Siliciano RF, Ho YC. 2017. Defective HIV-1 proviruses are expressed and can be recognized by cytotoxic T lymphocytes, which shape the proviral landscape. Cell Host Microbe 21:494–506. doi:10.1016/j.chom.2017.03.008 28407485 PMC5433942

[11] Cartwright EK, Spicer L, Smith SA, Lee D, Fast R, Paganini S, Lawson BO, Nega M, Easley K, Schmitz JE, Bosinger SE, Paiardini M, Chahroudi A, Vanderford TH, Estes JD, Lifson JD, Derdeyn CA, Silvestri G. 2016. CD8+ lymphocytes are required for maintaining viral suppression in SIV-infected macaques treated with short-term antiretroviral therapy. Immunity 45:656–668. doi:10.1016/j.immuni.2016.08.018 27653601 PMC5087330

[12] Kim Y, Anderson JL, Lewin SR. 2018. Getting the “Kill” into “Shock and Kill”: strategies to eliminate latent HIV. Cell Host Microbe 23:14–26. doi:10.1016/j.chom.2017.12.004 29324227 PMC5990418

[13] Ren Y, Huang SH, Patel S, Alberto WDC, Magat D, Ahimovic D, Macedo AB, Durga R, Chan D, Zale E, Mota TM, Truong R, Rohwetter T, McCann CD, Kovacs CM, Benko E, Wimpelberg A, Cannon C, Hardy WD, Bosque A, Bollard CM, Jones RB. 2020. BCL-2 antagonism sensitizes cytotoxic T cell-resistant HIV reservoirs to elimination ex vivo. J Clin Invest 130:2542–2559. doi:10.1172/JCI132374 32027622 PMC7191002

[14] Kuo HH, Ahmad R, Lee GQ, Gao C, Chen HR, Ouyang Z, Szucs MJ, Kim D, Tsibris A, Chun TW, Battivelli E, Verdin E, Rosenberg ES, Carr SA, Yu XG, Lichterfeld M. 2018. Anti-apoptotic protein BIRC5 maintains survival of HIV-1-infected CD4^+^ T cells. Immunity 48:1183–1194. doi:10.1016/j.immuni.2018.04.004 29802019 PMC6013384

[15] Cohn LB, da Silva IT, Valieris R, Huang AS, Lorenzi JCC, Cohen YZ, Pai JA, Butler AL, Caskey M, Jankovic M, Nussenzweig MC. 2018. Clonal CD4^+^ T cells in the HIV-1 latent reservoir display a distinct gene profile upon reactivation. Nat Med 24:604–609. doi:10.1038/s41591-018-0017-7 29686423 PMC5972543

[16] Chomont N, El-Far M, Ancuta P, Trautmann L, Procopio FA, Yassine-Diab B, Boucher G, Boulassel M-R, Ghattas G, Brenchley JM, Schacker TW, Hill BJ, Douek DC, Routy J-P, Haddad EK, Sékaly R-P. 2009. HIV reservoir size and persistence are driven by T cell survival and homeostatic proliferation. Nat Med 15:893–900. doi:10.1038/nm.1972 19543283 PMC2859814

[17] McMyn NF, Varriale J, Fray EJ, Zitzmann C, MacLeod H, Lai J, Singhal A, Moskovljevic M, Garcia MA, Lopez BM, et al.. 2023. The latent reservoir of inducible, infectious HIV-1 does not decrease despite decades of antiretroviral therapy. J Clin Invest 133:e171554. doi:10.1172/JCI171554 37463049 PMC10471168

[18] Cohn LB, Silva IT, Oliveira TY, Rosales RA, Parrish EH, Learn GH, Hahn BH, Czartoski JL, McElrath MJ, Lehmann C, Klein F, Caskey M, Walker BD, Siliciano JD, Siliciano RF, Jankovic M, Nussenzweig MC. 2015. HIV-1 integration landscape during latent and active infection. Cell 160:420–432. doi:10.1016/j.cell.2015.01.020 25635456 PMC4371550

[19] Lian X, Seiger KW, Parsons EM, Gao C, Sun W, Gladkov GT, Roseto IC, Einkauf KB, Osborn MR, Chevalier JM, Jiang C, Blackmer J, Carrington M, Rosenberg ES, Lederman MM, McMahon DK, Bosch RJ, Jacobson JM, Gandhi RT, Peluso MJ, Chun TW, Deeks SG, Yu XG, Lichterfeld M. 2023. Progressive transformation of the HIV-1 reservoir cell profile over two decades of antiviral therapy. Cell Host Microbe 31:83–96. doi:10.1016/j.chom.2022.12.002 36596305 PMC9839361

[20] Einkauf KB, Lee GQ, Gao C, Sharaf R, Sun X, Hua S, Chen SM, Jiang C, Lian X, Chowdhury FZ, Rosenberg ES, Chun TW, Li JZ, Yu XG, Lichterfeld M. 2019. Intact HIV-1 proviruses accumulate at distinct chromosomal positions during prolonged antiretroviral therapy. J Clin Invest 129:988–998. doi:10.1172/JCI124291 30688658 PMC6391088

[21] Einkauf KB, Osborn MR, Gao C, Sun W, Sun X, Lian X, Parsons EM, Gladkov GT, Seiger KW, Blackmer JE, Jiang C, Yukl SA, Rosenberg ES, Yu XG, Lichterfeld M. 2022. Parallel analysis of transcription, integration, and sequence of single HIV-1 proviruses. Cell 185:266–282. doi:10.1016/j.cell.2021.12.011 35026153 PMC8809251

[22] Kufera JT, Armstrong C, Wu F, Singhal A, Zhang H, Lai J, Wilkins HN, Simonetti FR, Siliciano JD, Siliciano RF. 2024. CD4+ T cells with latent HIV-1 have reduced proliferative responses to T cell receptor stimulation. J Exp Med 221:e20231511. doi:10.1084/jem.20231511 38270554 PMC10818065

[23] Hosmane NN, Kwon KJ, Bruner KM, Capoferri AA, Beg S, Rosenbloom DIS, Keele BF, Ho Y-C, Siliciano JD, Siliciano RF. 2017. Proliferation of latently infected CD4^+^ T cells carrying replication-competent HIV-1: Potential role in latent reservoir dynamics. J Exp Med 214:959–972. doi:10.1084/jem.20170193 28341641 PMC5379987

[24] Weinberger LS, Burnett JC, Toettcher JE, Arkin AP, Schaffer DV. 2005. Stochastic gene expression in a lentiviral positive-feedback loop: HIV-1 Tat fluctuations drive phenotypic diversity. Cell 122:169–182. doi:10.1016/j.cell.2005.06.006 16051143

[25] Hansen MMK, Wen WY, Ingerman E, Razooky BS, Thompson CE, Dar RD, Chin CW, Simpson ML, Weinberger LS. 2018. A post-transcriptional feedback mechanism for noise suppression and fate stabilization. Cell 173:1609–1621. doi:10.1016/j.cell.2018.04.005 29754821 PMC6044448

[26] Tantale K, Garcia-Oliver E, Robert MC, L’Hostis A, Yang Y, Tsanov N, Topno R, Gostan T, Kozulic-Pirher A, Basu-Shrivastava M, Mukherjee K, Slaninova V, Andrau JC, Mueller F, Basyuk E, Radulescu O, Bertrand E. 2021. Stochastic pausing at latent HIV-1 promoters generates transcriptional bursting. Nat Commun 12:4503. doi:10.1038/s41467-021-24462-5 34301927 PMC8302722

[27] Stern J, Solomon A, Dantanarayana A, Pascoe R, Reynaldi A, Davenport MP, Milush J, Deeks SG, Hartogensis W, Hecht FM, Cockerham L, Roche M, Lewin SR. 2022. Cell-associated human immunodeficiency virus (HIV) ribonucleic acid has a circadian cycle in males with HIV on antiretroviral therapy. J Infect Dis 225:1721–1730. doi:10.1093/infdis/jiab533 34655216 PMC9633724

[28] Chang CC, Naranbhai V, Stern J, Roche M, Dantanarayana A, Ke R, Tennakoon S, Solomon A, Hoh R, Hartogensis W, Hecht FM, Sikaris K, Price DJ, Elliott JH, Deeks SG, Churchill M, Cameron PU, Hengartner N, Perelson AS, Lewin SR. 2018. Variation in cell-associated unspliced HIV RNA on antiretroviral therapy is associated with the circadian regulator brain-and-muscle-ARNT-like-1. AIDS 32:2119–2128. doi:10.1097/QAD.0000000000001937 30005017 PMC6173794

[29] Borrmann H, Davies R, Dickinson M, Pedroza-Pacheco I, Schilling M, Vaughan-Jackson A, Magri A, James W, Balfe P, Borrow P, McKeating JA, Zhuang X. 2020. Pharmacological activation of the circadian component REV-ERB inhibits HIV-1 replication. Sci Rep 10:13271. doi:10.1038/s41598-020-70170-3 32764708 PMC7413328

[30] Ko CH, Takahashi JS. 2006. Molecular components of the mammalian circadian clock. Hum Mol Genet 15 Spec No 2:R271–R277. doi:10.1093/hmg/ddl207 16987893

[31] Zeichner SL, Kim JY, Alwine JC. 1991. Linker-scanning mutational analysis of the transcriptional activity of the human immunodeficiency virus type 1 long terminal repeat. J Virol 65:2436–2444. doi:10.1128/JVI.65.5.2436-2444.1991 2016766 PMC240597

[32] Guillaumond F, Dardente H, Giguère V, Cermakian N. 2005. Differential control of Bmal1 circadian transcription by REV-ERB and ROR nuclear receptors. J Biol Rhythms 20:391–403. doi:10.1177/0748730405277232 16267379

[33] Borrmann H, Ulkar G, Kliszczak AE, Ismed D, Schilling M, Magri A, Harris JM, Balfe P, Vasudevan S, Borrow P, Zhuang X, McKeating JA. 2023. Molecular components of the circadian clock regulate HIV-1 replication. iScience 26:107007. doi:10.1016/j.isci.2023.107007 37534138 PMC10391662

[34] Hu Y, Spengler ML, Kuropatwinski KK, Comas-Soberats M, Jackson M, Chernov MV, Gleiberman AS, Fedtsova N, Rustum YM, Gudkov AV, Antoch MP. 2011. Selenium is a modulator of circadian clock that protects mice from the toxicity of a chemotherapeutic drug via upregulation of the core clock protein, BMAL1. Oncotarget 2:1279–1290. doi:10.18632/oncotarget.411 22249125 PMC3282084

[35] Zerbato JM, Khoury G, Zhao W, Gartner MJ, Pascoe RD, Rhodes A, Dantanarayana A, Gooey M, Anderson J, Bacchetti P, Deeks SG, McMahon J, Roche M, Rasmussen TA, Purcell DFJ, Lewin SR. 2021. Multiply spliced HIV RNA is a predictive measure of virus production ex vivo and in vivo following reversal of HIV latency. EBioMedicine 65:103241. doi:10.1016/j.ebiom.2021.103241 33647768 PMC7920823

[36] Sundqvist KG. 2021. CD28 superagonist shock and blockage of motogenic T cell cascade. Front Immunol 12:670864. doi:10.3389/fimmu.2021.670864 33968078 PMC8098977

[37] Tarrado-Castellarnau M, Cortés R, Zanuy M, Tarragó-Celada J, Polat IH, Hill R, Fan TWM, Link W, Cascante M. 2015. Methylseleninic acid promotes antitumour effects via nuclear FOXO3a translocation through Akt inhibition. Pharmacol Res 102:218–234. doi:10.1016/j.phrs.2015.09.009 26375988 PMC4850087

[38] Hirota T, Kon N, Itagaki T, Hoshina N, Okano T, Fukada Y. 2010. Transcriptional repressor TIEG1 regulates Bmal1 gene through GC box and controls circadian clockwork. Genes Cells 15:111–121. doi:10.1111/j.1365-2443.2009.01371.x 20070857

[39] Igarashi T, Izumi H, Uchiumi T, Nishio K, Arao T, Tanabe M, Uramoto H, Sugio K, Yasumoto K, Sasaguri Y, Wang KY, Otsuji Y, Kohno K. 2007. Clock and ATF4 transcription system regulates drug resistance in human cancer cell lines. Oncogene 26:4749–4760. doi:10.1038/sj.onc.1210289 17297441

[40] Yang X, Xia R, Yue C, Zhai W, Du W, Yang Q, Cao H, Chen X, Obando D, Zhu Y, Chen X, Chen JJ, Piganelli J, Wipf P, Jiang Y, Xiao G, Wu C, Jiang J, Lu B. 2018. ATF4 regulates CD4^+^ T cell immune responses through metabolic reprogramming. Cell Rep 23:1754–1766. doi:10.1016/j.celrep.2018.04.032 29742431 PMC6051420

[41] Shi G, Xie P, Qu Z, Zhang Z, Dong Z, An Y, Xing L, Liu Z, Dong Y, Xu G, Yang L, Liu Y, Xu Y. 2016. Distinct roles of HDAC3 in the core circadian negative feedback loop are critical for clock function. Cell Rep 14:823–834. doi:10.1016/j.celrep.2015.12.076 26776516

[42] Guillaumond F, Gréchez-Cassiau A, Subramaniam M, Brangolo S, Peteri-Brünback B, Staels B, Fiévet C, Spelsberg TC, Delaunay F, Teboul M. 2010. Kruppel-like factor KLF10 is a link between the circadian clock and metabolism in liver. Mol Cell Biol 30:3059–3070. doi:10.1128/MCB.01141-09 20385766 PMC2876690

[43] Boivin DB, James FO, Wu A, Cho-Park PF, Xiong H, Sun ZS. 2003. Circadian clock genes oscillate in human peripheral blood mononuclear cells. Blood 102:4143–4145. doi:10.1182/blood-2003-03-0779 12893774

[44] Yu X, Rollins D, Ruhn KA, Stubblefield JJ, Green CB, Kashiwada M, Rothman PB, Takahashi JS, Hooper LV. 2013. TH17 cell differentiation is regulated by the circadian clock. Science 342:727–730. doi:10.1126/science.1243884 24202171 PMC4165400

[45] Kume K, Zylka MJ, Sriram S, Shearman LP, Weaver DR, Jin X, Maywood ES, Hastings MH, Reppert SM. 1999. mCRY1 and mCRY2 are essential components of the negative limb of the circadian clock feedback loop. Cell 98:193–205. doi:10.1016/s0092-8674(00)81014-4 10428031

[46] Preitner N, Damiola F, Lopez-Molina L, Zakany J, Duboule D, Albrecht U, Schibler U. 2002. The orphan nuclear receptor REV-ERBalpha controls circadian transcription within the positive limb of the mammalian circadian oscillator. Cell 110:251–260. doi:10.1016/s0092-8674(02)00825-5 12150932

[47] Canaple L, Rambaud J, Dkhissi-Benyahya O, Rayet B, Tan NS, Michalik L, Delaunay F, Wahli W, Laudet V. 2006. Reciprocal regulation of brain and muscle Arnt-like protein 1 and peroxisome proliferator-activated receptor alpha defines a novel positive feedback loop in the rodent liver circadian clock. Mol Endocrinol 20:1715–1727. doi:10.1210/me.2006-0052 16556735

[48] Oishi K, Shirai H, Ishida N. 2005. CLOCK is involved in the circadian transactivation of peroxisome-proliferator-activated receptor alpha (PPARalpha) in mice. Biochem J 386:575–581. doi:10.1042/BJ20041150 15500444 PMC1134877

[49] Asher G, Gatfield D, Stratmann M, Reinke H, Dibner C, Kreppel F, Mostoslavsky R, Alt FW, Schibler U. 2008. SIRT1 regulates circadian clock gene expression through PER2 deacetylation. Cell 134:317–328. doi:10.1016/j.cell.2008.06.050 18662546

[50] Nakahata Y, Kaluzova M, Grimaldi B, Sahar S, Hirayama J, Chen D, Guarente LP, Sassone-Corsi P. 2008. The NAD+-dependent deacetylase SIRT1 modulates CLOCK-mediated chromatin remodeling and circadian control. Cell 134:329–340. doi:10.1016/j.cell.2008.07.002 18662547 PMC3526943

[51] Masri S, Rigor P, Cervantes M, Ceglia N, Sebastian C, Xiao C, Roqueta-Rivera M, Deng C, Osborne TF, Mostoslavsky R, Baldi P, Sassone-Corsi P. 2014. Partitioning circadian transcription by SIRT6 leads to segregated control of cellular metabolism. Cell 158:659–672. doi:10.1016/j.cell.2014.06.050 25083875 PMC5472354

[52] Sun S, Liu Z, Feng Y, Shi L, Cao X, Cai Y, Liu B. 2019. Sirt6 deacetylase activity regulates circadian rhythms via Per2. Biochem Biophys Res Commun 511:234–238. doi:10.1016/j.bbrc.2019.01.143 30782483

[53] Miki T, Xu Z, Chen-Goodspeed M, Liu M, Van Oort-Jansen A, Rea MA, Zhao Z, Lee CC, Chang K-S. 2012. PML regulates PER2 nuclear localization and circadian function. EMBO J 31:1427–1439. doi:10.1038/emboj.2012.1 22274616 PMC3321181

[54] Onishi Y, Kawano Y. 2012. Rhythmic binding of Topoisomerase I impacts on the transcription of Bmal1 and circadian period. Nucleic Acids Res 40:9482–9492. doi:10.1093/nar/gks779 22904072 PMC3479213

[55] Druzd D, Matveeva O, Ince L, Harrison U, He W, Schmal C, Herzel H, Tsang AH, Kawakami N, Leliavski A, Uhl O, Yao L, Sander LE, Chen C-S, Kraus K, de Juan A, Hergenhan SM, Ehlers M, Koletzko B, Haas R, Solbach W, Oster H, Scheiermann C. 2017. Lymphocyte circadian clocks control lymph node trafficking and adaptive immune responses. Immunity 46:120–132. doi:10.1016/j.immuni.2016.12.011 28087238 PMC5263259

[56] Arnér ESJ. 2009. Focus on mammalian thioredoxin reductases--important selenoproteins with versatile functions. Biochim Biophys Acta 1790:495–526. doi:10.1016/j.bbagen.2009.01.014 19364476

[57] Shigemi Z, Manabe K, Hara N, Baba Y, Hosokawa K, Kagawa H, Watanabe T, Fujimuro M. 2017. Methylseleninic acid and sodium selenite induce severe ER stress and subsequent apoptosis through UPR activation in PEL cells. Chem Biol Interact 266:28–37. doi:10.1016/j.cbi.2017.01.027 28161410

[58] Evans SO, Jacobson GM, Goodman HJB, Bird S, Jameson MB. 2020. Comparison of three oral selenium compounds in cancer patients: evaluation of differential pharmacodynamic effects in normal and malignant cells. J Trace Elem Med Biol 58:126446. doi:10.1016/j.jtemb.2019.126446 31838377

[59] Varlamova EG, Goltyaev MV, Turovsky EA. 2022. The role of selenoproteins SELENOM and SELENOT in the regulation of apoptosis, ER stress, and calcium homeostasis in the A-172 human glioblastoma cell line. Biology (Basel) 11:811. doi:10.3390/biology11060811 35741332 PMC9220170

[60] Telwatte S, Lee S, Somsouk M, Hatano H, Baker C, Kaiser P, Kim P, Chen TH, Milush J, Hunt PW, Deeks SG, Wong JK, Yukl SA. 2018. Gut and blood differ in constitutive blocks to HIV transcription, suggesting tissue-specific differences in the mechanisms that govern HIV latency. PLoS Pathog 14:e1007357. doi:10.1371/journal.ppat.1007357 30440043 PMC6237391

[61] Yukl SA, Kaiser P, Kim P, Telwatte S, Joshi SK, Vu M, Lampiris H, Wong JK. 2018. HIV latency in isolated patient CD4^+^ T cells may be due to blocks in HIV transcriptional elongation, completion, and splicing. Sci Transl Med 10:eaap9927. doi:10.1126/scitranslmed.aap9927 29491188 PMC5959841

[62] Borrmann H, Ismed D, Kliszczak AE, Borrow P, Vasudevan S, Jagannath A, Zhuang X, McKeating JA. 2023. Inhibition of salt inducible kinases reduces rhythmic HIV-1 replication and reactivation from latency. J Gen Virol 104:001877. doi:10.1099/jgv.0.001877 37529926 PMC10721046

[63] Wang Z, Jiang C, Lü J. 2002. Induction of caspase-mediated apoptosis and cell-cycle G1 arrest by selenium metabolite methylselenol. Mol Carcinog 34:113–120. doi:10.1002/mc.10056 12112305

[64] Li Z, Carrier L, Rowan BG. 2008. Methylseleninic acid synergizes with tamoxifen to induce caspase-mediated apoptosis in breast cancer cells. Mol Cancer Ther 7:3056–3063. doi:10.1158/1535-7163.MCT-07-2142 18790785

[65] Hattori SI, Matsuda K, Tsuchiya K, Gatanaga H, Oka S, Yoshimura K, Mitsuya H, Maeda K. 2018. Combination of a latency-reversing agent with a smac mimetic minimizes secondary HIV-1 infection in vitro. Front Microbiol 9:2022. doi:10.3389/fmicb.2018.02022 30283406 PMC6156138

[66] Campbell GR, Bruckman RS, Chu YL, Trout RN, Spector SA. 2018. SMAC mimetics induce autophagy-dependent apoptosis of HIV-1-infected resting memory CD4+ T cells. Cell Host Microbe 24:689–702. doi:10.1016/j.chom.2018.09.007 30344003 PMC6250054

[67] Qiu C, Zhang T, Zhu X, Qiu J, Jiang K, Zhao G, Wu H, Deng G. 2019. Methylseleninic acid suppresses breast cancer growth via the JAK2/STAT3 pathway. Reprod Sci 26:829–838. doi:10.1177/1933719118815582 30526368

[68] Mavigner M, Liao LE, Brooks AD, Ke R, Mattingly C, Schoof N, McBrien J, Carnathan D, Liang S, Vanderford TH, Paiardini M, Kulpa D, Lifson JD, Dunham RM, Easley KA, Margolis DM, Perelson AS, Silvestri G, Chahroudi A, Simon V. 2021. CD8 lymphocyte depletion enhances the latency reversal activity of the SMAC mimetic AZD5582 in ART-suppressed SIV-infected rhesus macaques. J Virol 95:e01429-20. doi:10.1128/JVI.01429-20 33568515 PMC8103677

[69] López-Huertas MR, Mateos E, Sánchez Del Cojo M, Gómez-Esquer F, Díaz-Gil G, Rodríguez-Mora S, López JA, Calvo E, López-Campos G, Alcamí J, Coiras M. 2013. The presence of HIV-1 Tat protein second exon delays fas protein-mediated apoptosis in CD4+ T lymphocytes: a potential mechanism for persistent viral production. J Biol Chem 288:7626–7644. doi:10.1074/jbc.M112.408294 23364796 PMC3597804

[70] Berro R, de la Fuente C, Klase Z, Kehn K, Parvin L, Pumfery A, Agbottah E, Vertes A, Nekhai S, Kashanchi F. 2007. Identifying the membrane proteome of HIV-1 latently infected cells. J Biol Chem 282:8207–8218. doi:10.1074/jbc.M606324200 17237230

[71] Timilsina U, Gaur R. 2016. Modulation of apoptosis and viral latency - an axis to be well understood for successful cure of human immunodeficiency virus. J Gen Virol 97:813–824. doi:10.1099/jgv.0.000402 26764023

[72] Laurent-Crawford AG, Krust B, Rivière Y, Desgranges C, Muller S, Kieny MP, Dauguet C, Hovanessian AG. 1993. Membrane expression of HIV envelope glycoproteins triggers apoptosis in CD4 cells. AIDS Res Hum Retroviruses 9:761–773. doi:10.1089/aid.1993.9.761 8105835

[73] Chen D, Wang M, Zhou S, Zhou Q. 2002. HIV-1 Tat targets microtubules to induce apoptosis, a process promoted by the pro-apoptotic Bcl-2 relative Bim. EMBO J 21:6801–6810. doi:10.1093/emboj/cdf683 12486001 PMC139103

[74] Vallejo-Gracia A, Chen IP, Perrone R, Besnard E, Boehm D, Battivelli E, Tezil T, Krey K, Raymond KA, Hull PA, Walter M, Habrylo I, Cruz A, Deeks S, Pillai S, Verdin E, Ott M. 2020. FOXO1 promotes HIV latency by suppressing ER stress in T cells. Nat Microbiol 5:1144–1157. doi:10.1038/s41564-020-0742-9 32541947 PMC7483895

[75] Mendoza P, Jackson JR, Oliveira TY, Gaebler C, Ramos V, Caskey M, Jankovic M, Nussenzweig MC, Cohn LB. 2020. Antigen-responsive CD4+ T cell clones contribute to the HIV-1 latent reservoir. J Exp Med 217:e20200051. doi:10.1084/jem.20200051 32311008 PMC7336300

[76] Simonetti FR, Zhang H, Soroosh GP, Duan J, Rhodehouse K, Hill AL, Beg SA, McCormick K, Raymond HE, Nobles CL, Everett JK, Kwon KJ, White JA, Lai J, Margolick JB, Hoh R, Deeks SG, Bushman FD, Siliciano JD, Siliciano RF. 2021. Antigen-driven clonal selection shapes the persistence of HIV-1-infected CD4+ T cells in vivo. J Clin Invest 131:e145254. doi:10.1172/JCI145254 33301425 PMC7843227

[77] Lennicke C, Rahn J, Bukur J, Hochgräfe F, Wessjohann LA, Lichtenfels R, Seliger B. 2017. Modulation of MHC class I surface expression in B16F10 melanoma cells by methylseleninic acid. Oncoimmunology 6:e1259049. doi:10.1080/2162402X.2016.1259049 28680742 PMC5486188

[78] Nair D, Rådestad E, Khalkar P, Diaz-Argelich N, Schröder A, Klynning C, Ungerstedt J, Uhlin M, Fernandes AP. 2018. Methylseleninic acid sensitizes ovarian cancer cells to T-cell mediated killing by decreasing PDL1 and VEGF levels. Front Oncol 8:407. doi:10.3389/fonc.2018.00407 30324091 PMC6172341

[79] Ghosh A, Khanam A, Ray K, Mathur P, Subramanian A, Poonia B, Kottilil S. 2023. CD38: an ecto-enzyme with functional diversity in T cells. Front Immunol 14:1146791. doi:10.3389/fimmu.2023.1146791 37180151 PMC10172466

[80] Camacho-Pereira J, Tarragó MG, Chini CCS, Nin V, Escande C, Warner GM, Puranik AS, Schoon RA, Reid JM, Galina A, Chini EN. 2016. CD38 dictates age-related NAD decline and mitochondrial dysfunction through an SIRT3-dependent mechanism. Cell Metab 23:1127–1139. doi:10.1016/j.cmet.2016.05.006 27304511 PMC4911708

[81] Lee J, Kang R, Park S, Saliu IO, Son M, Voorhees JR, Dimitry JM, Quillin EI, Woodie LN, Lananna BV, Gan L, Goo Y-A, Zhao G, Lazar MA, Burris TP, Musiek ES. 2025. REV-ERBα regulates brain NAD+ levels and tauopathy via an NFIL3–CD38 axis. Nat Aging 5:2070–2085. doi:10.1038/s43587-025-00950-x 40890338 PMC12532575

[82] Campbell GR, To RK, Zhang G, Spector SA. 2020. SMAC mimetics induce autophagy-dependent apoptosis of HIV-1-infected macrophages. Cell Death Dis 11:590. doi:10.1038/s41419-020-02761-x 32719312 PMC7385130

[83] Arandjelovic P, Kim Y, Cooney JP, Preston SP, Doerflinger M, McMahon JH, Garner SE, Zerbato JM, Roche M, Tumpach C, Ong J, Sheerin D, Smyth GK, Anderson JL, Allison CC, Lewin SR, Pellegrini M. 2023. Venetoclax, alone and in combination with the BH3 mimetic S63845, depletes HIV-1 latently infected cells and delays rebound in humanized mice. Cell Rep Med 4:101178. doi:10.1016/j.xcrm.2023.101178 37652018 PMC10518630

[84] Muzembo BA, Ngatu NR, Januka K, Huang H-L, Nattadech C, Suzuki T, Wada K, Ikeda S. 2019. Selenium supplementation in HIV-infected individuals: a systematic review of randomized controlled trials. Clin Nutr ESPEN 34:1–7. doi:10.1016/j.clnesp.2019.09.005 31677697

[85] Marshall JR, Burk RF, Ondracek RP, Hill KE, Perloff M, Davis W, Pili R, George S, Bergan R. 2017. Selenomethionine and methyl selenocysteine: multiple-dose pharmacokinetics in selenium-replete men. Oncotarget 8:26312–26322. doi:10.18632/oncotarget.15460 28412747 PMC5432259

[86] Ip C, Thompson HJ, Zhu Z, Ganther HE. 2000. In vitro and in vivo studies of methylseleninic acid: evidence that a monomethylated selenium metabolite is critical for cancer chemoprevention. Cancer Res 60:2882–2886. 10850432

[87] Marshall JR, Ip C, Romano K, Fetterly G, Fakih M, Jovanovic B, Perloff M, Crowell J, Davis W, French-Christy R, Dew A, Coomes M, Bergan R. 2011. Methyl selenocysteine: single-dose pharmacokinetics in men. Cancer Prev Res (Phila) 4:1938–1944. doi:10.1158/1940-6207.CAPR-10-0259 21846796 PMC3208773

[88] Balsalobre A, Brown SA, Marcacci L, Tronche F, Kellendonk C, Reichardt HM, Schütz G, Schibler U. 2000. Resetting of circadian time in peripheral tissues by glucocorticoid signaling. Science 289:2344–2347. doi:10.1126/science.289.5488.2344 11009419

[89] Tamayo AG, Shukor S, Burr A, Erickson P, Parekkadan B. 2020. Tracking leukemic T-cell transcriptional dynamics in vivo with a blood-based reporter assay. FEBS Open Bio 10:1868–1879. doi:10.1002/2211-5463.12940 PMC7459418 32710494

[90] Ghosh D. 1992. Glucocorticoid receptor-binding site in the human immunodeficiency virus long terminal repeat. J Virol 66:586–590. doi:10.1128/JVI.66.1.586-590.1992 1727502 PMC238321

[91] Markham PD, Salahuddin SZ, Veren K, Orndorff S, Gallo RC. 1986. Hydrocortisone and some other hormones enhance the expression of HTLV-III. Int J Cancer 37:67–72. doi:10.1002/ijc.2910370112 3000956

[92] Jones KA, Kadonaga JT, Luciw PA, Tjian R. 1986. Activation of the AIDS retrovirus promoter by the cellular transcription factor, Sp1. Science 232:755–759. doi:10.1126/science.3008338 3008338

[93] Harrich D, Garcia J, Wu F, Mitsuyasu R, Gonazalez J, Gaynor R. 1989. Role of SP1-binding domains in in vivo transcriptional regulation of the human immunodeficiency virus type 1 long terminal repeat. J Virol 63:2585–2591. doi:10.1128/JVI.63.6.2585-2591.1989 2657100 PMC250732

[94] Bender H, Wang Z, Schuster N, Krieglstein K. 2004. TIEG1 facilitates transforming growth factor-beta-mediated apoptosis in the oligodendroglial cell line OLI-neu. J Neurosci Res 75:344–352. doi:10.1002/jnr.10856 14743447

[95] Turrini F, Marelli S, Kajaste-Rudnitski A, Lusic M, Van Lint C, Das AT, Harwig A, Berkhout B, Vicenzi E. 2015. HIV-1 transcriptional silencing caused by TRIM22 inhibition of Sp1 binding to the viral promoter. Retrovirology (Auckl) 12:104. doi:10.1186/s12977-015-0230-0 PMC4683785 26683615

[96] Jiang G, Santos Rocha C, Hirao LA, Mendes EA, Tang Y, Thompson GR III, Wong JK, Dandekar S. 2017. HIV exploits antiviral host innate GCN2-ATF4 signaling for establishing viral replication early in infection. mBio 8. doi:10.1128/mBio.01518-16 PMC5414007 28465428

[97] Neill G, Masson GR. 2023. A stay of execution: ATF4 regulation and potential outcomes for the integrated stress response. Front Mol Neurosci 16:1112253. doi:10.3389/fnmol.2023.1112253 36825279 PMC9941348

[98] Yang Z, Kim H, Ali A, Zheng Z, Zhang K. 2017. Interaction between stress responses and circadian metabolism in metabolic disease. Liver Res 1:156–162. doi:10.1016/j.livres.2017.11.002 29430321 PMC5805151

[99] Bollinger T, Bollinger A, Skrum L, Dimitrov S, Lange T, Solbach W. 2009. Sleep-dependent activity of T cells and regulatory T cells. Clin Exp Immunol 155:231–238. doi:10.1111/j.1365-2249.2008.03822.x 19040608 PMC2675254

[100] Bollinger T, Leutz A, Leliavski A, Skrum L, Kovac J, Bonacina L, Benedict C, Lange T, Westermann J, Oster H, Solbach W. 2011. Circadian clocks in mouse and human CD4+ T cells. PLoS One 6:e29801. doi:10.1371/journal.pone.0029801 22216357 PMC3247291

[101] Nobis CC, Dubeau Laramée G, Kervezee L, Maurice De Sousa D, Labrecque N, Cermakian N. 2019. The circadian clock of CD8 T cells modulates their early response to vaccination and the rhythmicity of related signaling pathways. Proc Natl Acad Sci USA 116:20077–20086. doi:10.1073/pnas.1905080116 31527231 PMC6778233

[102] Fortier EE, Rooney J, Dardente H, Hardy MP, Labrecque N, Cermakian N. 2011. Circadian variation of the response of T cells to antigen. J Immunol 187:6291–6300. doi:10.4049/jimmunol.1004030 22075697

[103] Stincardini C, Pariano M, D’Onofrio F, Renga G, Orecchini E, Orabona C, Nunzi E, Gargaro M, Fallarino F, Chun SK, Fortin BM, Masri S, Brancorsini S, Romani L, Costantini C, Bellet MM. 2023. The circadian control of tryptophan metabolism regulates the host response to pulmonary fungal infections. PNAS Nexus 2:gad036. doi:10.1093/pnasnexus/pgad036 PMC9991457 36896128

[104] de Assis LVM, Kinker GS, Moraes MN, Markus RP, Fernandes PA, Castrucci AM de L. 2018. Expression of the circadian clock gene BMAL1 positively correlates with antitumor immunity and patient survival in metastatic melanoma. Front Oncol 8:185. doi:10.3389/fonc.2018.00185 29946530 PMC6005821

[105] Yang Y, Yuan G, Xie H, Wei T, Zhu D, Cui J, Liu X, Shen R, Zhu Y, Yang X. 2019. Circadian clock associates with tumor microenvironment in thoracic cancers. Aging (Albany NY) 11:11814–11828. doi:10.18632/aging.102450 31881010 PMC6949103

[106] Dashti HS, Jones SE, Wood AR, Lane JM, van Hees VT, Wang H, Rhodes JA, Song Y, Patel K, Anderson SG, et al.. 2019. Genome-wide association study identifies genetic loci for self-reported habitual sleep duration supported by accelerometer-derived estimates. Nat Commun 10:1100. doi:10.1038/s41467-019-08917-4 30846698 PMC6405943

[107] Popescu A, Ottaway C, Ford K, Medina E, Patterson TW, Ingiosi A, Hicks SC, Singletary K, Peixoto L. 2025. Transcriptional dynamics of sleep deprivation and subsequent recovery sleep in the male mouse cortex. Physiol Genomics 57:431–445. doi:10.1152/physiolgenomics.00128.2024 40315180 PMC12140865

[108] Pan T, Song Z, Wu L, Liu G, Ma X, Peng Z, Zhou M, Liang L, Liu B, Liu J, Zhang J, Zhang X, Huang R, Zhao J, Li Y, Ling X, Luo Y, Tang X, Cai W, Deng K, Li L, Zhang H. 2019. USP49 potently stabilizes APOBEC3G protein by removing ubiquitin and inhibits HIV-1 replication. eLife 8. doi:10.7554/eLife.48318 PMC6701944 31397674

[109] Jordan A, Bisgrove D, Verdin E. 2003. HIV reproducibly establishes a latent infection after acute infection of T cells in vitro. EMBO J 22:1868–1877. doi:10.1093/emboj/cdg188 12682019 PMC154479

[110] Clouse KA, Powell D, Washington I, Poli G, Strebel K, Farrar W, Barstad P, Kovacs J, Fauci AS, Folks TM. 1989. Monokine regulation of human immunodeficiency virus-1 expression in a chronically infected human T cell clone. J Immunol 142:431–438. 2463307

[111] Saleh S, Solomon A, Wightman F, Xhilaga M, Cameron PU, Lewin SR. 2007. CCR7 ligands CCL19 and CCL21 increase permissiveness of resting memory CD4+ T cells to HIV-1 infection: a novel model of HIV-1 latency. Blood 110:4161–4164. doi:10.1182/blood-2007-06-097907 17881634

[112] Elliott JH, Wightman F, Solomon A, Ghneim K, Ahlers J, Cameron MJ, Smith MZ, Spelman T, McMahon J, Velayudham P, et al.. 2014. Activation of HIV transcription with short-course vorinostat in HIV-infected patients on suppressive antiretroviral therapy. PLoS Pathog 10:e1004473. doi:10.1371/journal.ppat.1004473 25393648 PMC4231123

[113] Chen Y, Chen L, Lun ATL, Baldoni PL, Smyth GK. 2025. edgeR v4: powerful differential analysis of sequencing data with expanded functionality and improved support for small counts and larger datasets. Nucleic Acids Res 53. doi:10.1093/nar/gkaf018 PMC11754124 39844453

[114] Rainer J, Gatto L, Weichenberger CX. 2019. Ensembldb: an R package to create and use Ensembl-based annotation resources. Bioinformatics 35:3151–3153. doi:10.1093/bioinformatics/btz031 30689724 PMC6736197

[115] Wu T, Hu E, Xu S, Chen M, Guo P, Dai Z, Feng T, Zhou L, Tang W, Zhan L, Fu X, Liu S, Bo X, Yu G. 2021. clusterProfiler 4.0: a universal enrichment tool for interpreting omics data. The Innovation 2:100141. doi:10.1016/j.xinn.2021.100141 34557778 PMC8454663

