# Supplementary material for: Methaneseleninic acid, a circadian-modulating agent, reactivates latent HIV-1 infection without cellular activation or proliferation
Source: J Virol. 2026 Feb 25;100(3):e01983-25. doi: 10.1128/jvi.01983-25 (PMC13011396; doi:10.1128/jvi.01983-25)
Supplement: Supplemental material — Table S1; Fig. S1 to S10. [file jvi.01983-25-s0002.docx]

**Supplementary Tables**

**Supp. Table 1**

Supplementary Table 1 – Summary of circadian-modulating compounds screened for HIV-1 latency reversal properties in J-Lat Tat-IRES-GFP A2 cells, and their mechanism of action on circadian cycles as described in the literature. Relative half-maximal lethal concentrations (LC_50_) and effective concentrations (EC_50_) were calculated from dose-response curves fitted to data collected at 48h. The effects on circadian cycles were identified in references in the literature.

| Compound | Effect on Circadian Cycle | Ref | LC_50_  (95%  Confidence  Interval) | EC_50_  (95%  Confidence  Interval) | Maximal GFP %  Expression  (Median,  (range)) | Concentration  of Maximal GFP %  Expression |
| --- | --- | --- | --- | --- | --- | --- |
| Nobiletin | ↑ ROR transcription | (1-4) | N/A | N/A | <1 % | N/A |
| Resveratrol | ↑ CLOCK: BMAL1 stability | (5) | 279.6 μM  (219.8 - 361.7) | 35.1 μM  (15.3 - 172.6) | 31.1 %  (26.2 – 37.8) | 100 μM |
| Ivermectin | ↓ amplitude,  ↑ period length | (6) | 4.6 μM  (2.42 – 8.61) | 0.015 μM  (0.0015 – 0.13) | 35.3 %  (19.5 - 38.3) | 2.5 μM |
| Methyl-selenocysteine | ↑ BMAL1 transcription | (7) | 63.3 μM  (37.3 – 105.2) | 78.4 μM  (64.4 – 95.2) | 78.5 %  (74.8 - 81.1) | 1000 μM |
| MSA | ↑ BMAL1 transcription | (7) | 2.7uM  (0.33 – 23.2) | 0.044 μM  (0.011 – 0.15) | 70.1 %  (69.4 - 70.2) | 10 μM |

**Supplementary Figures**

**Supp. Figure 1**

**
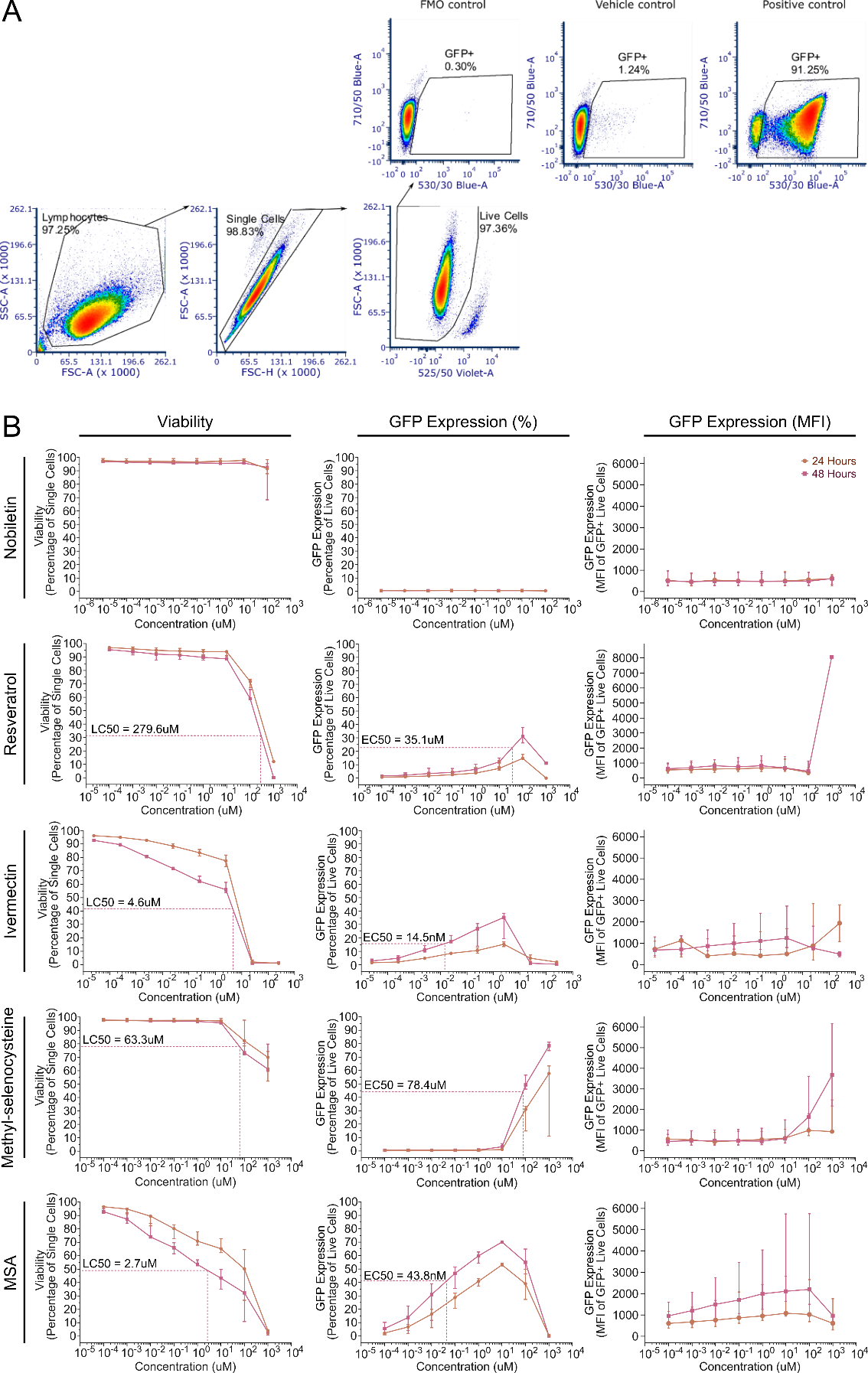
**

**Supplementary Figure 1 –Assessment of circadian-modulating compound latency reversal in latently infected cell lines**. A) Gating strategy for flow cytometric analysis of drug toxicity and latency reversal in J-Lat Tat-IRES-GFP A2 cells. B) Dose response curves of drug toxicity and latency reversal in J-Lat Tat-IRES-GFP A2 cells at 24 (orange) and 48 hours (pink). n=3, datapoints represent the median ±range. Relative half-maximal lethal concentrations (LC50) and effective concentrations (EC50) were calculated from dose-response curves fitted to data collected at 48h (pink dashed line). MFI, Median Fluorescence Intensity.

**Supp. Figure 2**

**
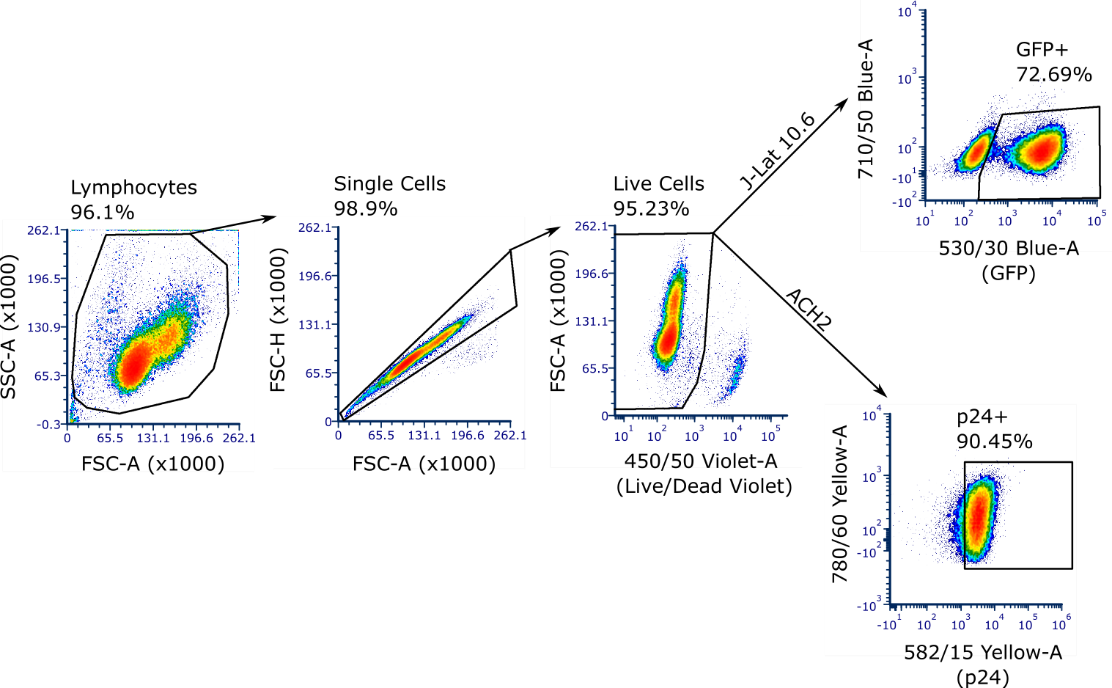
**

**Supplementary Figure 2 – Gating strategy for flow cytometric assessment of viability and latency reversal in J-Lat 10.6 and ACH2 cell lines.** J-Lat 10.6 and ACH2 cells (lymphocytes) were discriminated in size from cell debris by forward-scatter (FSC) and side-scatter (SSC) area (A), then singlets were gated based on FSC-A and FSC-height (H). Live cells were identified by staining with the Fixable Live/Dead Cell Death Stain Violet in the 450/50 Violet bandpass. Of the live cells, GFP expression in J-Lat 10.6 cells was detected in the 530/30 Blue bandpass, and p24 expression in ACH2 cells detected in the 582/15 Yellow bandpass.

**Supp. Figure 3**
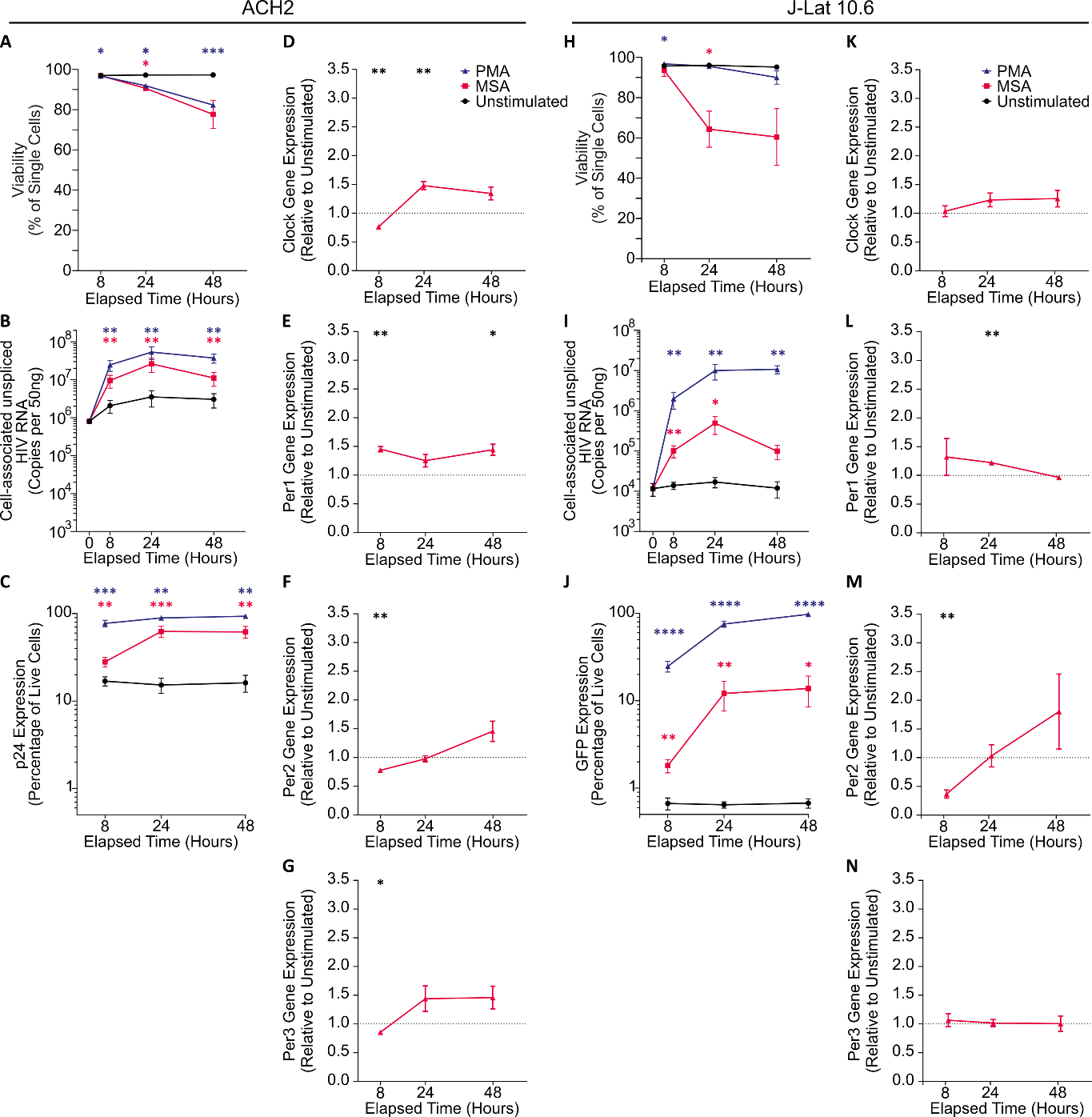


**Supplementary Figure 3- MSA reactivates HIV-1 and induces Period gene transcription in latently infected T-cell lines.** ACH2 (A-G) and J-Lat 10.6 (H-N) cells were stimulated with 10uM MSA (red) for 18 hours, at which point the drug was washed off and cells cultured for a further 30 hours. Cells left unstimulated (black) or stimulated with 16nM phorbol 12-myristate 13-acetate (PMA) and 500uM ionomycin (blue) provided negative and positive controls for viral reactivation, respectively. Cell viability (A, H) was assessed by flow cytometry, along with viral protein expression (C, J) Cell-associated unspliced HIV-1 RNA (B, I) was measured by RT-qPCR. Circadian gene expression (D-G, K-N) was measured by RT-qPCR and fold-changes calculated compared to baseline using the ΔΔCt relative quantification method, with Gapdh as a reference gene. Datapoints represent the mean±SEM. Statistical significance was calculated by paired t-tests; p<0.05, *; p<0.01, **; p<0.001, ***; p<0.0001, ****

**Supp Figure 4**

**
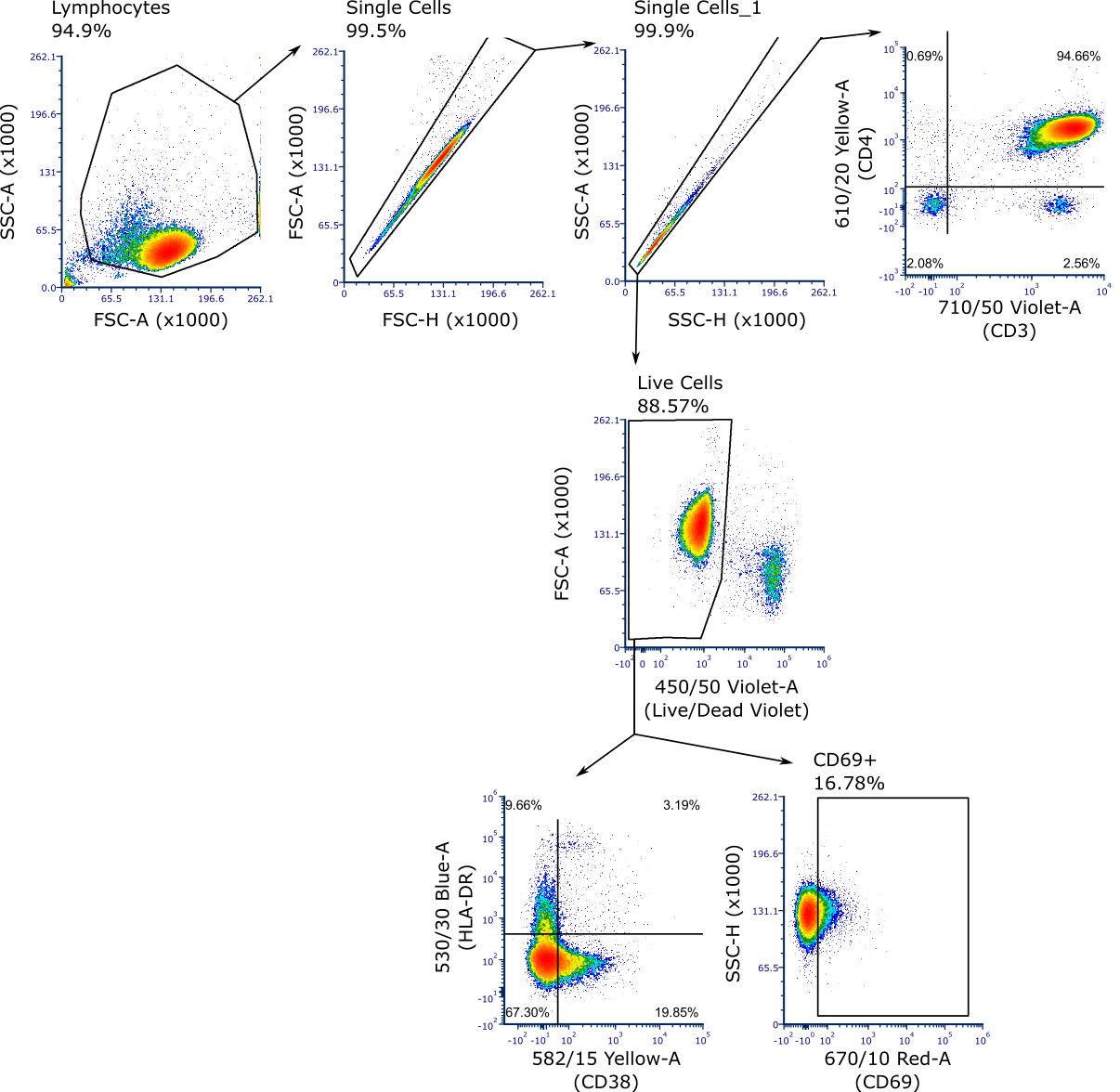
**

**Supplementary Figure 4 - Flow Cytometry Gating Strategy for Assessment of MSA Stimulation of Primary CD4+ T-cells from People Living with HIV.** Primary CD4+ T-cells from PWH (lymphocytes) were discriminated in size from cell debris by forward-scatter (FSC) and side-scatter (SSC) area (A), then singlets were gated on based on FSC-A and FSC-height (H), and SSC-A and SSC-H. Purity of isolated cells was assessed by co-expression of CD3 and CD4, measured as fluorescence in the 710/50 Violet and 610/20 Yellow bandpasses, respectively. Live cells were identified by staining with the Fixable Live/Dead Cell Death Stain Violet in the 450/50 Violet bandpass. Of the live cells, activation markers CD38, HLA-DR and CD69 were measured as fluorescence in the 582/15 Yellow, 530/30 Blue and 670/10 Red bandpasses, respectively

**Supp Figure 5**


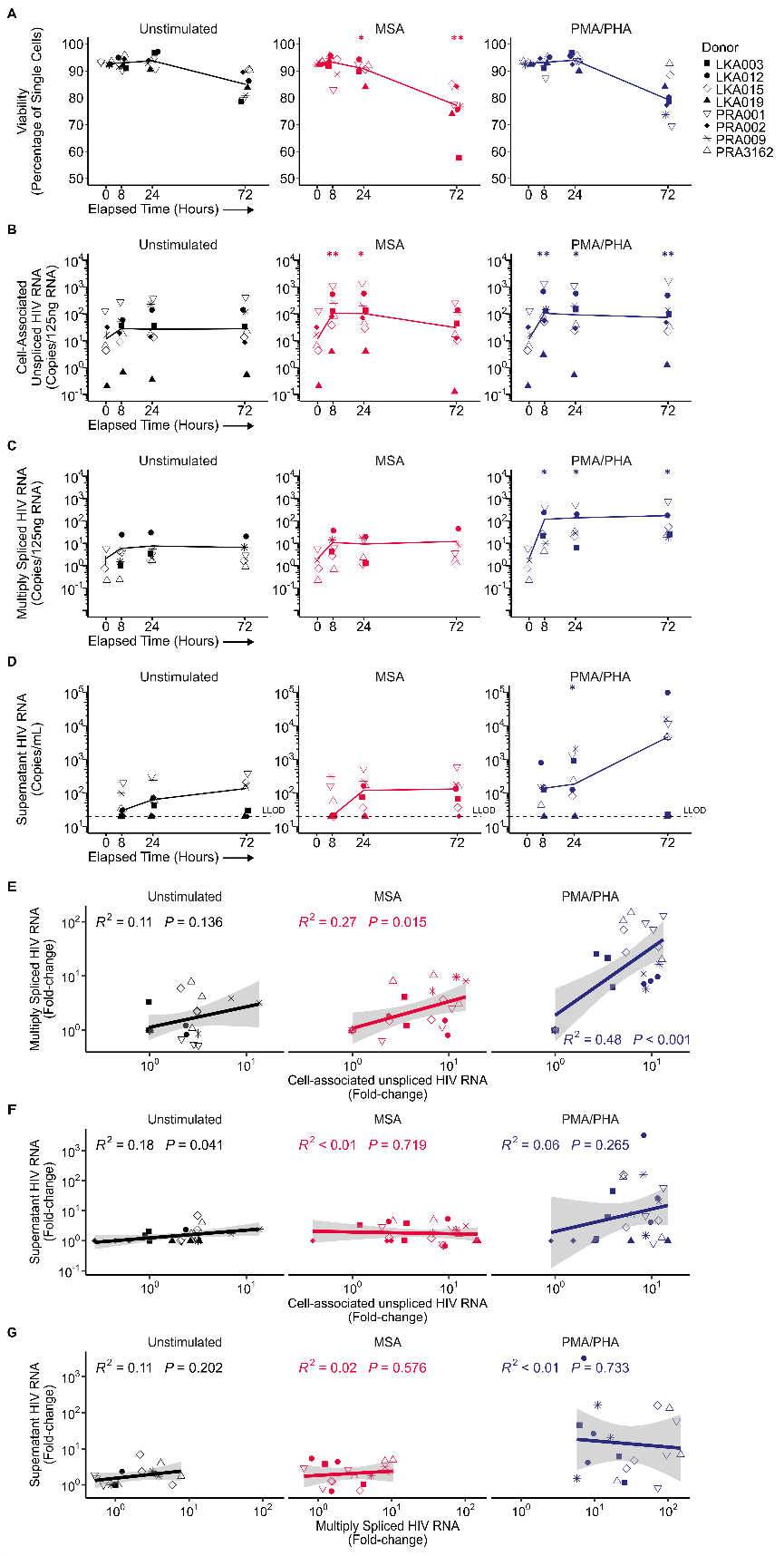


**Supplementary Figure 5 - MSA Potently Induces Expression of Cell-associated Unspliced HIV-1.** CD4+ T-cells were isolated from PBMCs from PWH and cultured in the presence of 10uM methaneseleninic acid (MSA, red) for 24 hours before drug wash off, or continuous culture with 10nM phorbol 12-myristate 13-acetate (PMA) and 10ug/mL phytohaemagglutinin (PHA) (blue). Viability (A) was assessed by flow cytometry, cell-associated unspliced (B) and multiply spliced (C) HIV-1 RNA was quantified from bulk RNA and supernatant HIV-1 RNA (D) quantified by RT-qPCR. n=8 independent donors, datapoints represent individual participants and lines represent the median. Statistical compared to unstimulated cells was determined using a Wilcoxon matched-pairs signed rank test; p<0.05, *; p<0.01, **. Linear regression of log-transformed fold-changes between cell-associated unspliced HIV RNA and multiply spliced HIV RNA (E), cell-associated unspliced HIV RNA and supernatant HIV RNA (F), multiply spliced HIV RNA and supernatant HIV RNA (G). Datapoints represent individual participants, solid lines represent the linear regression line fitted to data, and grey areas represent the 95% confidence interval.

**Supp Figure 6**


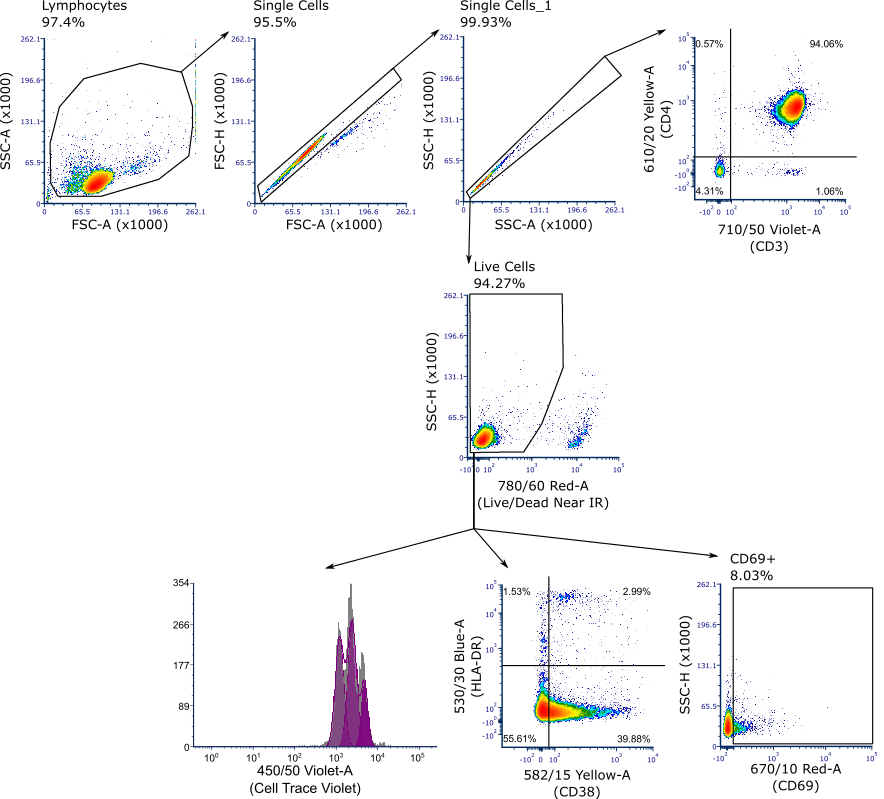


**Supplementary Figure 6 – Flow Cytometry Gating Strategy for Assessment of MSA Stimulation of HIV-uninfected Primary CD4+ T-cells.** HIV-uninfected primary CD4+ T-cells (lymphocytes) were discriminated in size from cell debris by forward-scatter (FSC) and side-scatter (SSC) area (A), then singlets were gated on based on FSC-A and FSC-height (H), and SSC-A and SSC-H. Purity of isolated cells was assessed by co-expression of CD3 and CD4, measured as fluorescence in the 710/50 Violet and 610/20 Yellow bandpasses, respectively. Live cells were identified by staining with the Fixable Live/Dead Cell Death Stain Near Infrared (IR) in the 780/60 Red bandpass. Of the live cells, proliferation was measured based on CellTrace Violet staining 109 in the 450/50 Violet bandpass. Activation markers CD38, HLA-DR and CD69 of live cells were measured as fluorescence in the 582/15 Yellow, 530/30 Blue and 670/10 Red bandpasses, respectively.

**Supp Figure 7
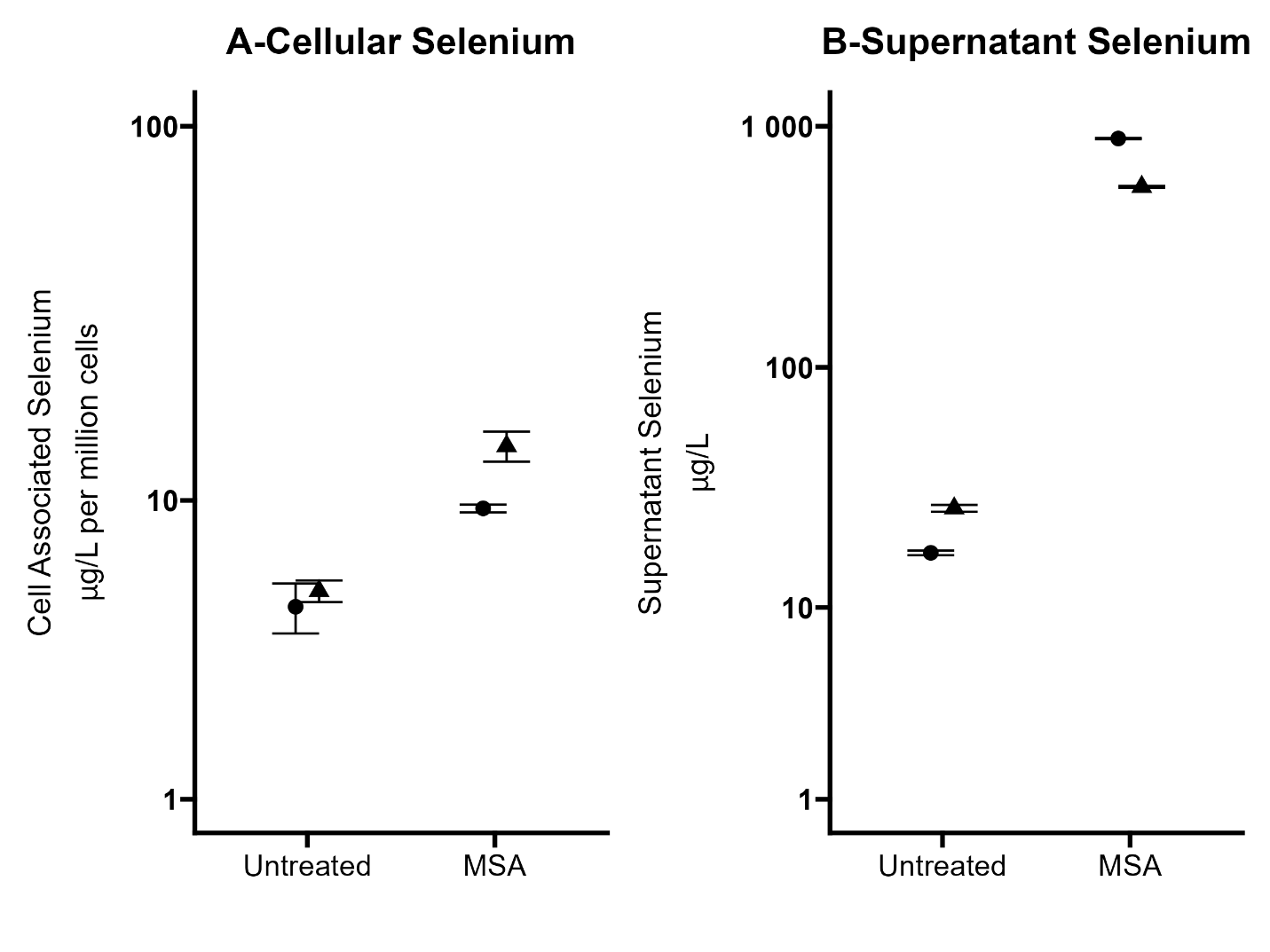
**

**Supplementary Figure 7 – MSA treatment of CD4+ T cells leads to an increase in cell associated selenium.** A) Selenium proportion in cells and, B) supernatant post treatment of HIV-uninfected primary CD4+ T-cells with MSA. Data are representative of the sum of the Se isotopes (Se78 and Se80). Graphs are presented as median and standard deviation of technical replicates of two (circle, triangle) independent experiments.

**Supp Figure 8**

**
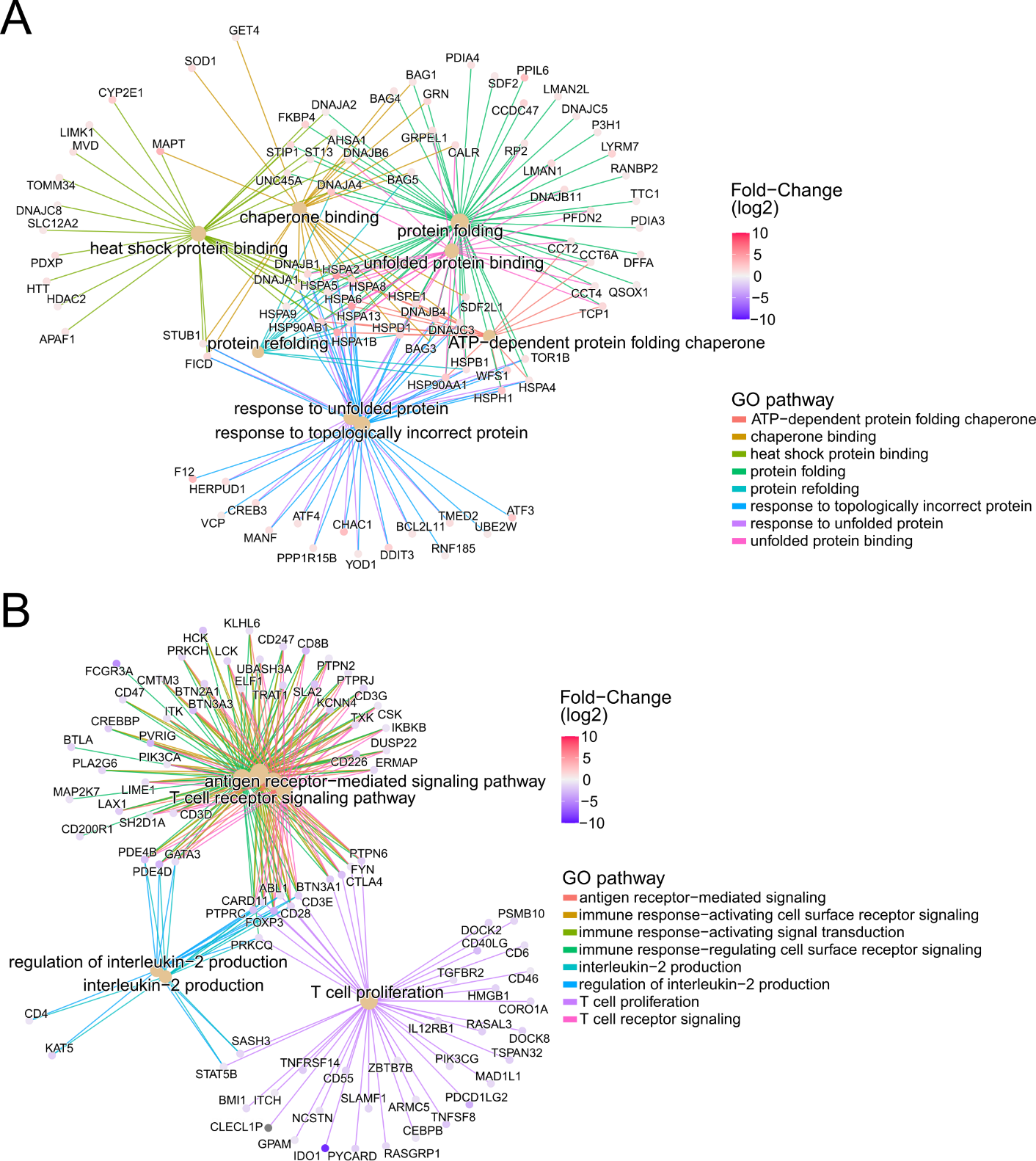
**

**Supplementary Figure 8 – MSA Induces an Unfolded Protein Response and Inhibits T-cell Proliferation.**  Cluster network analysis of upregulated (A) and downregulated (B) Gene Ontology (GO) pathways 8 hours post-MSA treatment. Top dysregulated GO pathways are depicted by beige circles, and the genes contributing to their dysregulation with circles depicting the log_2_ fold-change at 8 hours compared to baseline.

**Supp Figure 9**

**
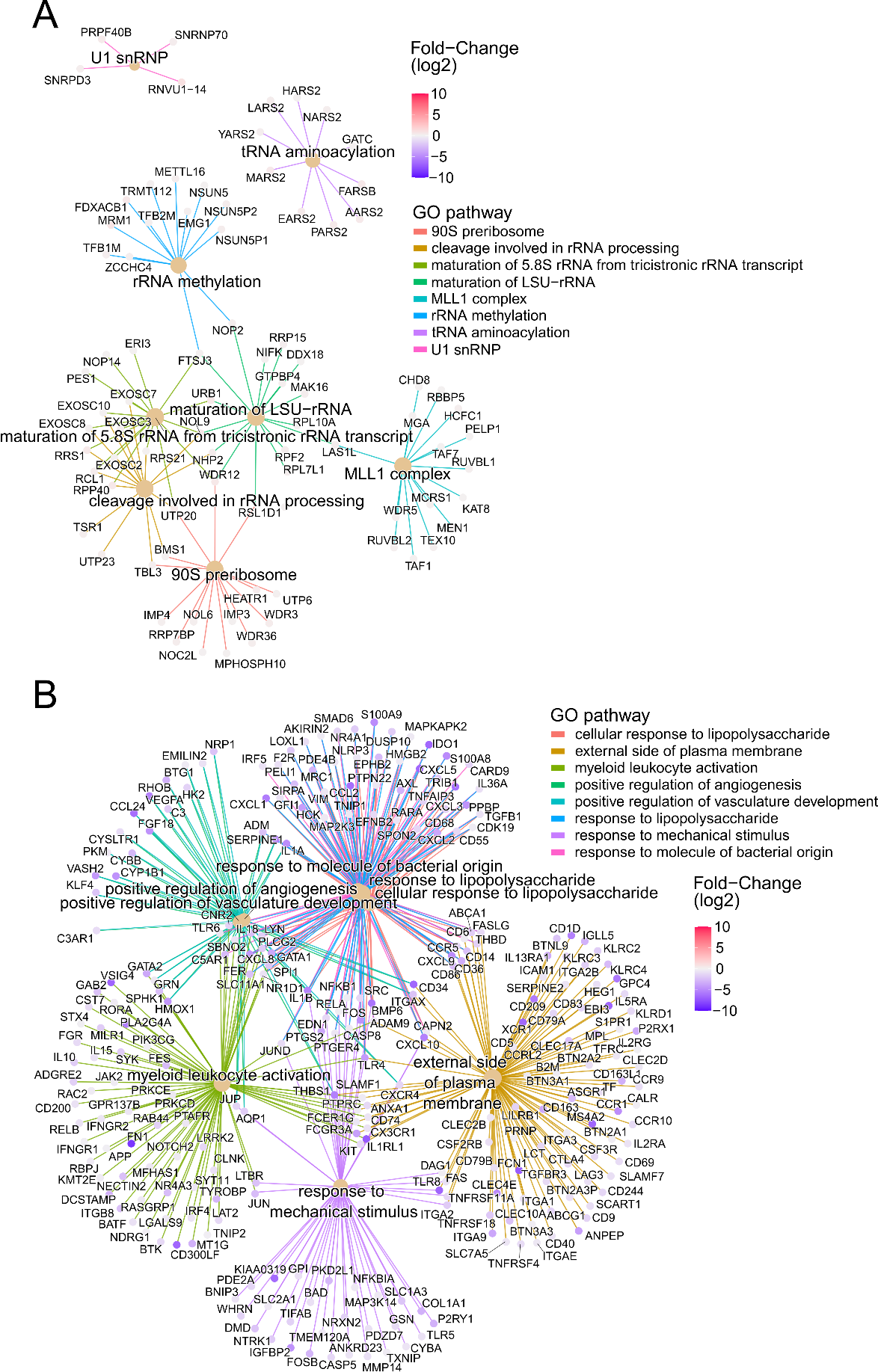
**

**Supplementary Figure 9 - MSA Inhibits Late Cellular Activation and Chemokine Signalling.** Cluster network analysis of upregulated (A) and downregulated (B) Gene Ontology (GO) pathways 72 hours post-MSA treatment. Top dysregulated GO pathways are depicted by beige circles, and the genes contributing to their dysregulation with circles depicting the log_2_ fold-change at 72 hours compared to baseline

**Supp Figure 10**


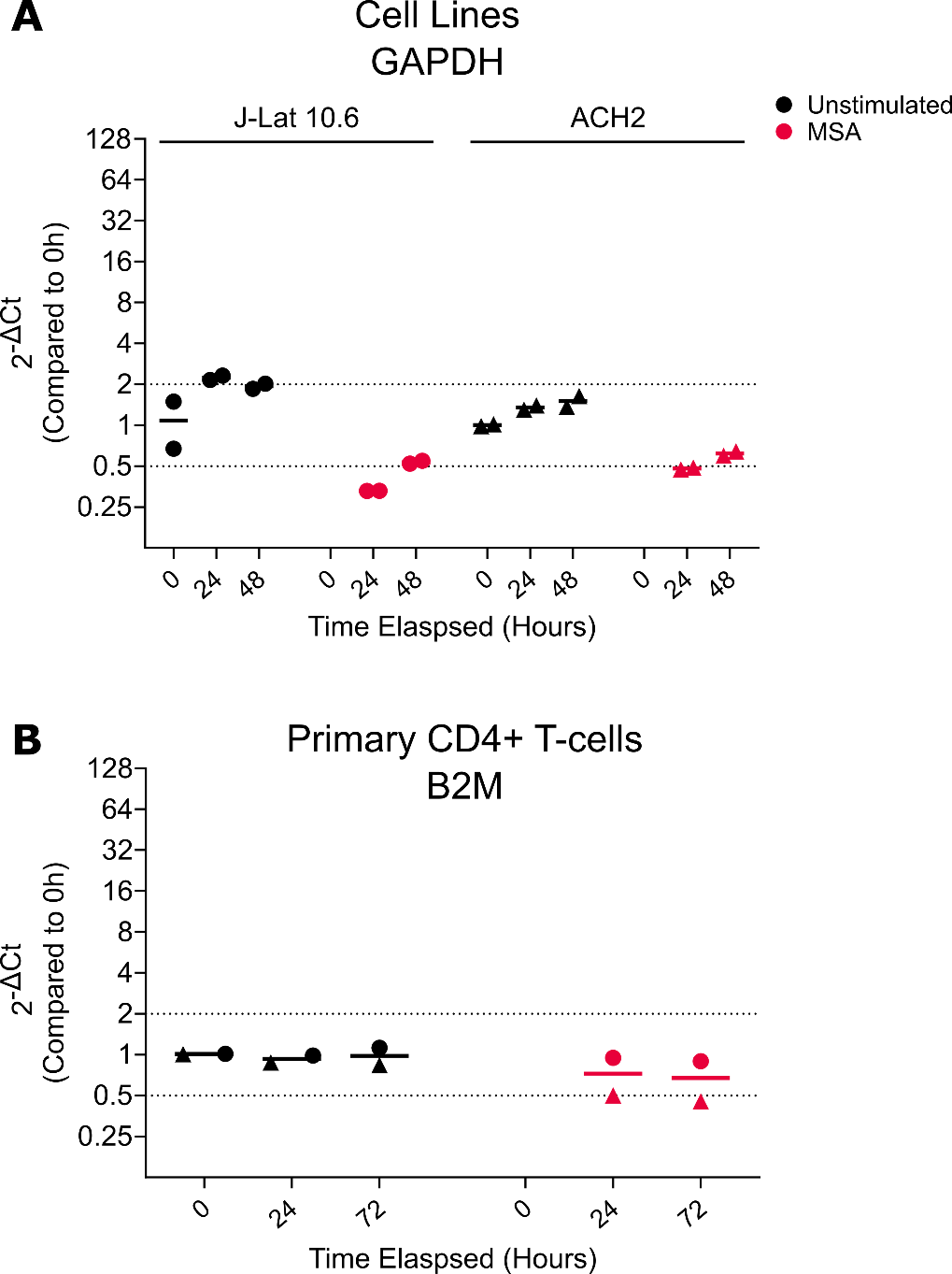


**Supplementary Figure 10 – Expression of housekeeping genes, GAPDH and B2M, remains stable over time in vitro .** mRNA levels of the housekeeping gene, GAPDH (A) and B2M (B), were measured by RT-qPCR over time in cultures of HIV-infected cell lines, and primary CD4+ T-cells from HIV-negative donors, respectively. Expression levels are quantified using the ΔCt method and presented as fold-changes (2^-ΔCt^) in unstimulated (black) or MSA-stimulated (red) cells. Means are represented as a line and individual datapoints represent experimental replicates (A) or independent participants (B).
